# Coprecipitation of Ce(III) oxide with UO_2_

**DOI:** 10.1107/S1600577524008336

**Published:** 2024-09-30

**Authors:** M. Saleh, M. Hedberg, P. L. Tam, K. Spahiu, I. Persson, C. Ekberg

**Affiliations:** a Nuclear Chemistry / Industrial Materials Recycling Chalmers University of Technology SE-412 96Gothenburg Sweden; b Department of Industrial and Materials Science Chalmers University of Technology SE-412 96Gothenburg Sweden; c Swedish Nuclear Fuel and Waste Management Co. SE-101 24Stockholm Sweden; d Swedish University of Agricultural Sciences (SLU) Uppsala SE-750 07 Sweden; ESRF – The European Synchrotron, France

**Keywords:** UO_2_, Ce (III), coprecipitation, solid solution, solubility, amorphous, actinides

## Abstract

The neutralization of acidic solutions containing U (IV) and Ce (III) at room temperature in glove box atmosphere and in the presence of di­thio­nite results in coprecipitation of these elements as amorphous solid solutions Ce_*x*_U_1–*x*_O_2±*y*_. The solubilities of the precipitates with different mole fractions (*x*) of Ce(OH)_3_ (*x* = 0.01 or 0.1) were determined in 1 *M* NaClO_4_ solutions between pH 2.2 and 12.8 under reducing conditions. The solids were investigated by a variety of methods (chemical analysis, SEM-EDX, XRD, XPS, XAS) to determine the nature of the solid solutions formed, their composition and the valence state of Ce and U. X-ray photoelectron spectroscopy confirmed the oxidation states of the solids both before and after the equilibration as Ce (III) and U (IV). The amorphous coprecipitates reached equilibrium relatively fast (∼1 week). The release of Ce from the coprecipitates was totally dominated by the release of uranium over the whole pH range. The Ce concentrations decrease slightly with the decrease of Ce content in the solid, suggesting that Ce_*x*_U_1–*x*_O_2±*y*_ solids behave thermodynamically as solid solutions. The concentrations of U in equilibrium with the coprecipitate were in excellent agreement with the solubility of UO_2_(s) under reducing conditions reported in the literature. The conditional solubility product of Ce(OH)_3_ from the coprecipitate was several orders of magnitude (∼4 in the near neutral pH range and ∼18 in the acidic range) lower than that of pure Ce(OH)_3_(s). The activities and activity coefficients of Ce(OH)_3_(s) in the coprecipitate were also estimated. Activity coefficients are much less than 1, indicating that the mixing of Ce(OH)_3_ with UO_2_ is highly favorable.

## Abstract 2

Neutralization of acidic solutions containing U (IV) and Ce (III) at room temperature in glove box atmosphere and in the presence of di­thio­nite results in coprecipitation of these elements as amorphous solid solutions Ce_*x*_U_1–*x*_O_2±*y*_. The solids were investigated by a variety of methods to determine the nature of the solid solutions formed, their composition and the valence state of Ce and U.

## 1. Introduction

The leaching of the used nuclear fuel is, as the source term, one of the pivotal phenomena in the safety analysis of an underground repository for spent nuclear fuel. This fuel is mainly UO_2_(s) and thus highly insoluble in deep groundwaters, which are anoxic and reducing. The dissolution of the stored nuclear fuel in a future deep repository is a very complex process and is caused mainly by the radioactivity of the fuel itself. Ionizing radiation causes ionization or bond breaking of water molecules (radiolysis), producing both oxidants and reductants in similar amounts. For kinetic reasons, molecular oxidants will predominantly react with the fuel causing its oxidative dissolution (Jonsson *et al.*, 2007 ▸). It is well known that dissolved molecular hydrogen, which is usually inert under repository temperatures, contributes in consuming part of the oxidants in solution through its reaction with the OH radical,
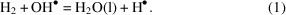
Recent studies have shown that the effects noticed in tests of spent fuel leaching under hydrogen atmosphere are much stronger and mainly due to processes occurring at the fuel surface (Cui *et al.*, 2008 ▸). Despite the multiple observations of the effect of hydrogen in the oxidative dissolution of the spent fuel and alpha-doped UO_2_, the only explanation offered presently is based on the effect of the metallic ɛ-particles present in spent fuel. The effect of these particles has been already thoroughly investigated (Broczkowski *et al.*, 2005 ▸; Trummer *et al.*, 2009 ▸) and their interaction with dissolved H_2_ has been used in models which predict the absence of oxidative dissolution for fuel older than 100 years in the presence of only 0.1 bar H_2_ (Jonsson *et al.*, 2007 ▸).

In most of the spent fuel leaching tests carried out in the presence of hydrogen, a decrease in the concentrations of uranium and other actinides such as Np and Pu is observed in the first days to weeks of the test, and afterwards they remain very low and constant during several months to years. This has led most of the authors to conclude that these actinide elements, originating from a pre-oxidized fuel layer, are reduced and precipitate as the corresponding reduced amorphous oxides. Their constant concentrations during months or years afterwards were interpreted as due to equilibrium with the corresponding reduced state amorphous oxides, *i.e.* UO_2_(am), NpO_2_(am) and PuO_2_(am) or Pu(OH)_3_(am). In some of these publications (Spahiu *et al.*, 2004 ▸; Cui *et al.*, 2008 ▸; Fors *et al.*, 2009 ▸; Ekeroth *et al.*, 2020 ▸; Puranen *et al.*, 2020 ▸) it has been observed that the measured concentrations of Np and Pu in solution are much lower than what is predicted from thermodynamic equilibrium data (Guillaumont *et al.*, 2003 ▸; Grenthe *et al.*, 2020 ▸). NpO_2_(am) and UO_2_(am) have very similar solubilities, about 3 × 10^−9^ *M* and 10^−9^ *M*, respectively; thus similar concentrations of Np and U would be expected if they are at equilibrium with the corresponding amorphous oxides. This is not the case in fuel leaching tests under hydrogen: Np is about three orders of magnitude lower than U. Further, the concentrations of Np and Pu in solution are lower than the concentration in equilibrium with the corresponding reduced oxide by the same factor as their concentration is lower than that of uranium in spent nuclear fuel. In spent fuel there is usually ∼95% U, ∼0.9% Pu and <0.1% Np and the observed Pu and Np concentrations are ∼100 and 1000 times, respectively, lower than ∼10^9^ *M*, which is the measured concentration of uranium. The possibility of these actinide ions coprecipitate with each other is high, given the similarity of their ionic radii with eightfold coordination: 0.96 Å for Pu (IV), 0.98 Å for Np (IV) and 1.00 Å for U (IV), as well as 1.0 Å for Pu (III) with sixfold coordination (Shannon, 1976 ▸), and of the fluorite type structures of their tetravalent oxides. The solubility of Pu (IV) oxide is 10^−10.8^ *M* and could explain the plutonium data by equilibrium with pure PuO_2_(s), but we find it unexpected that U (IV) coprecipitates with Np (IV) and not with Pu (IV).Thermodynamics predicts that this is possible only if a regular solid solution of the oxides of U, Pu and Np is formed, containing them in the proportions 100:1:0.1, *i.e.* as their content in the spent fuel. It is therefore important to investigate whether a solid solution of these actinide oxides can be formed during spent fuel leaching and to determine whether the solid solution is regular. In the case of irregular solid solution, the concentration of the minor component can decrease much more than what corresponds to its proportion in the solid, due to the influence of solid-state activity coefficients. This is why a study of the coprecipitation of U, Pu and Np in waters which simulate repository conditions needs to be undertaken.

Coprecipitation was first suggested as a radionuclide retention mechanism from spent fuel by Bruno *et al.* (Bruno *et al.*, 1985 ▸; Bruno & Sandino, 1987 ▸, 1988 ▸), much earlier than the first studies of fuel leaching under hydrogen were started. The coprecipitation was expected to occur when the uranium precipitated as UO_2_(s) under the reducing conditions of the far field. Bruno and co-workers investigated the coprecipitation of UO_2_(s) with Ln (III), Th (IV), Ba (II) and Pu (III). Despite the importance of such a mechanism, which can significantly lower the solubility of the minor component, only a few other studies have been carried out at room temperature with amorphous oxides. Among these are the study of Rousseau *et al.* (2002 ▸) who have investigated the coprecipitation of thorium with UO_2_, Sass & Rai (1987 ▸) who have investigated amorphous Cr (III)–Fe (III) hydroxide coprecipitation, and Rai *et al.* (2004 ▸) on the coprecipitation of amorphous UO_2_ with NpO_2_.

In order to investigate the basic principles and fine tune the technique, the coprecipitation of U (IV) and Ce (III) was studied, where Ce (III) was used to simulate Pu (III). The Ce (III) cation has an effective radius of 1.03 Å (sixfold coordination), quite similar to that of Pu (III). Under the repository conditions, both Pu (III) and Pu (IV) are expected to exist (Neck *et al.*, 2007 ▸) but in our case the choice of Ce (III) is determined by the need to protect U (IV) from oxidation by a strong reductant, which makes it difficult to use Ce (IV). The coprecipitated solid contains cations of different valence, U (IV) and Ce (III), so some kind of charge compensation is necessary. In a study of U (IV)–Pu (III) oxides prepared from thermal decomposition of the oxalate precursor, Arab-Chapelet *et al.* (2008 ▸) discuss replacement of U (IV) by Pu (III) in the oxalate structure and charge compensation by monovalent cations. For Pu (III) content up to 29 mol%, no excess oxygen was necessary in the oxide structure, *i.e.* the solid was U_0.71_Pu_0.29_O_2_(s). According to Kleykamp (1993 ▸), Ce_2_O_3_(s) is completely miscible with UO_2_(s) given that the uranium vacancies in stoichiometric UO_2_(s) are favorable sites for the solution of fission products such as Ce (Grimes & Catlow, 1991 ▸). In a study of U–La solid solutions, Prieur *et al.* (2018 ▸) point out that the O/*M* ratio in U_1–*y*_*M*_*y*_O_2±*x*_(s) solids solutions depends on the oxygen partial pressure during sintering, and it cannot be properly determined without experimental measurements. However, they point out that a few studies report an O/*M* ratio of 2.00. We carried out the synthesis of our coprecipitates in the presence of a reductant maintaining uranium as U (IV), which is possible only for very low oxygen fugacities (<10^−65^ atm; Rai *et al.*, 1990 ▸). We have no information about charge compensation in our coprecipitated solids, so we refer to them as Ce_0.01_U_0.99_ solid or coprecipitate instead of (Ce_0.01_U_0.99_)O_2±*x*_(s).

The objectives of this study were to determine whether the coprecipitation of actinides oxides or the formation of solid solutions can occur during spent fuel leaching under disposal conditions and the nature of solid solutions formed. For this purpose, amorphous solid solutions Ce_*x*_U_1–*x*_O_2±*y*_ were prepared, characterized both before and after equilibration in 1 *M* NaClO_4_ solutions by using several experimental techniques, while the total concentrations of Ce and U in solution were determined using inductively coupled plasma mass spectrometry (ICP-MS).

## 2. Methods and materials

### 2.1. Chemicals

All solutions used in the experimental work were prepared from ultrapure water with a resistivity of 18.2 MΩ cm (MilliQ Advantage, Merck) which was thoroughly sparged with N_2_ (99.99%) for several hours to remove any trace amounts of dissolved O_2_, transferred into an Ar atmosphere control chamber (glove box) and kept sealed in a glass container before use.

Uranium (IV) stock solution (∼111 g L^−1^) was prepared by dissolving a reactor grade uranium metallic rod (Norway) in 12 *M* HCl (37% ACS reagent, Sigma-Aldrich, Merck).

The concentrated HCl acidic solution was cooled prior to the dissolution of the U metal rod due to the exothermic reaction resulting from the dissolution process
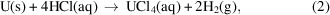
and was heated in the later stages when the reaction slowed down. The hot dissolved U (IV) solution was then filtered via vacuum filtration using a glass frit filter (G4) to remove any particles of U(s) or UO_2_(s) present in the solution. After the vacuum filtration, the filtrate was centrifuged (Beckman Coulter Avanti j-26 SXP centrifuge) to separate any solid particles from the solution. After centrifugation, the solution was filtered using a syringe filter and 0.45 µm polypropyl­ene membrane previously activated. Finally, the filtered U (IV) stock solution was transferred into an opaque glass bottle with glass frit stopcock and sealed with parafilm. The U stock solution had ∼1.8 *M* HCl in excess and was analyzed for U and acidity a few weeks after preparation (see the supporting information), so that any particles <0.45 µm would have dissolved. The following precautions were taken during the preparation of the U (IV) stock solution and during the equilibrations to avoid the presence of any oxidized uranium species or dissolved oxygen:

(1) Treating the uranium (IV) stock solution with uranium fine particles before each use to eliminate any oxidized uranium species.

(2) Storing the uranium stock solution in an opaque tightly closed glass container inside the glovebox.

(3) Filling the sample tubes to nearly maximum capacity to minimize gas space.

(4) Enclosing the samples in a closed glass container or vessel containing FeSO_4_ as an oxygen trap.

Several chemical analyses (spectrophotometric analysis, gravimetric analysis, Gran titration) were carried out to determine the concentration, acidity and oxidation state of the uranium stock solution before use. Details of these analyses can be found in the supporting information.

A 0.125 *M* Ce stock solution (∼18 g L^−1^) was prepared by dissolving 4.6573 g of CeCl_3_.7H_2_O of 99.9% purity (Sigma-Aldrich, Merck) in 100 ml of ∼10 *M* HCl (37% ACS reagent, Sigma-Aldrich, Merck). The solution had high acidity to simulate an available Pu (IV) stock solution. Sodium di­thio­nite, Na_2_S_2_O_4_ (Sigma-Aldrich, Merck), was used to prepare 10 m*M* and 20 m*M* stock solution of Na_2_S_2_O_4_.

1 *M* NaOH titrisol ampoules (Sigma-Aldrich, Merck) was used to prepare 1 *M* stock solution of carbonate-free NaOH. For the preparation of the ionic medium used for the solubility experiment, a 4.61308 mmol g^−1^ solution of NaClO_4_ stock solution was prepared from reagent grade perchloric acid and sodium carbonate following the laboratory methods developed at KTH (KTH, 1959 ▸). The salt concentration of the stock solution was determined by weighing samples dried at 125°C while the H^+^ concentration was analyzed via Gran plots (Gran, 1952 ▸). To prepare the 1 *M* NaClO_4_ solution, a calculated amount of stock solution containing 1 mol NaClO_4_ (216.77 g) was weighed in a 1 L volumetric flask and the flask was filled to the mark with distilled water.

### 2.2. Experimental procedure

All experiments were conducted in a glovebox with Ar atmosphere (Inert Technology) (99.99% Ar with ≤1 p.p.m. O_2_). The glovebox atmosphere is continuously circulated past a catalytic bed that removes O_2_, maintaining a level of ≤1 p.p.m. throughout the experiments. The glovebox was operated at ambient temperature (21.0 ± 2.0°C).

An aliquot of the acidic U (IV) stock solution for the coprecipitation experiment was initially treated with uranium grains (a few mg) for some minutes before transferring into an empty 250 ml centrifuge tube in order to reduce any potential traces of U (VI). To the centrifuge tube containing the U (IV) solution, a 20 m*M* de­oxy­genated solution of Na_2_S_2_O_4_ was added in order to maintain the reducing conditions.

A calculated aliquot corresponding to 1% acidic Ce (III) stock solution was added to the 250 ml centrifuge tube containing both U (IV) solution and 20 m*M* Na_2_S_2_O_4_.

Upon addition in the tube, the sample solution in the tube was slowly titrated with carbonate-free NaOH to precipitate U and Ce as amorphous hydroxides. The carbonate-free NaOH solution was added stepwise to the 250 ml centrifuge tube until a pH value of 9.5–10 was obtained in the neutralized solution. The presence of any carbonate in the NaOH solution would not affect the results, because the calculated NaOH volume was used only as a guide; we continued adding NaOH until reaching the target pH of the neutralized solution.

Black precipitates formed quickly on all occasions. The 250 ml centrifuge tube containing the precipitated solid was placed on an orbital shaker to allow for continued mixing for 15 min at 170 r.p.m. The solid solution was then centrifuged at 12000 r.p.m. (*g* value 12865) for 15 min (25°C) using a Beckman Coulter Avanti j-26 SXP centrifuge. After centrifugation, the supernatant was separated from the solid coprecipitates.

The resulting precipitate was washed twice with de­oxy­genated 20 m*M* Na_2_S_2_O_4_ solution adjusted to pH 7 in order to remove the NaCl formed during neutralization. Finally, the washed precipitate was aged overnight in 120 ml of 20 m*M* Na_2_S_2_O_4_ solution adjusted to pH 7 and the centrifuge tube was placed on the orbital shaker to have a continuous mixing of the solid and solution.

After aging, the formed slurry was centrifuged at 12000 r.p.m. for 15 min. The supernatant was removed as before, leaving behind the solid. Portions of the solids (a few mg) were distributed in different 50 ml Oak Ridge centrifuge tubes (Thermo Scientific) to be used for solubility measurements. Solutions containing 0.980 *M* NaClO_4_ and 10 m*M* Na_2_S_2_O_4_ were used as the ionic medium for the solubility measurements. The pH range for the solubility measurement was between 2 and 13 and was adjusted by adding carbonate-free NaOH or HClO_4_ in droplets prior to the solid addition in the tube. The pH of the solutions was measured before and after addition of the solid using a combined glass pH electrode calibrated against pH buffers.

Two sets of experiments were conducted with these precipitates. One set of experiments was conducted with 1% molar concentration of Ce and the other with 10% Ce concentration.

To the rest of the slurry or solid remaining in the 250 ml centrifuge tube, 75 ml of 10 m*M* sodium di­thio­nite was added to the tube to wash the precipitate. The solid, together with the added Na_2_S_2_O_4_ solution, was centrifuged at 12000 r.p.m. for 10 min and the removed supernatant was tested for chloride with AgNO_3_. The washing step was repeated twice. During the first wash, white precipitate forms, when the removed supernatant was tested with AgNO_3_, which indicates the presence of chloride. The precipitate after washing for the second time was left with little or no chloride. Then after a final washing step, the precipitate was washed once with 50 ml ultrapure water and, after centrifugation, the supernatant was tested for chloride indicating no presence of chloride. The solid precipitate after the final washing steps was left to dry in the glove box for some days before any further analysis of the solids.

The pH of the samples for the solubility experiments were measured at each equilibration period of 7, 14, 21 and 30 days using a combined glass pH electrode calibrated against pH buffers. The 3 *M* KCl reference solution of the combined pH glass electrode was replaced with a 3 *M* NaCl solution in order to avoid precipitation of KClO_4_, and the electrode was calibrated with pH buffers of pH 1 (HCl), 4 (biphtalate), 7 (phosphate) and 10 (KCl/H_3_BO_3_/NaOH) from Sigma Aldrich. The −log[H^+^] value in the 1 *M* NaClO_4_ solutions was calculated through the relationship: pH_exp_ + log[H^+^] = −0.23 ± 0.2 (Fanghänel *et al.*, 1996 ▸).

We made some attempts to measure the redox potential with a commercial combined Pt electrode after changing the reference compartment composition and did not obtain reliable values due to large electrode drift and erratic behavior. That is why we chose not to report them, even though the values obtained all corresponded to reducing conditions.

The solid suspensions in the different centrifuge tubes were continuously shaken using an orbital shaker until they were analyzed. At each equilibration period, an aliquot of the sample solutions was withdrawn from each tube and used for analysis.

To effectively separate tiny solid particles from solution, the sample solution from each tube was withdrawn using a syringe filter and 0.20 µm polypropyl­ene membrane. At a final separation step, Amicon Ultra-4 centrifugal filters with 30000 molecular weight cut-offs (NMWL Sigma Aldrich Merck, Millipore Ltd) were used to further separate the solid from the solution. The filters were pretreated with an aliquot of the sample which was passed through the filter and discarded to avoid any sorption losses. The filtered samples were subjected to various analytical methods.

### 2.3. Solution analysis for U and Ce by ICP-MS

The filtered solution samples were analyzed in triplicates for total U and Ce concentration with an ICP-MS instrument (Thermo Scientific iCAP Q). The measurements were performed in standard modes. The solution samples were diluted with 0.5 *M* HNO_3_ (Suprapur, Merck) containing 2 p.p.b. Bi-209 as an internal standard [from a 10 p.p.m. certified standard stock solution (CPAchem)].

The external calibration series of the analyzed elements in the concentration range of 0–50 p.p.b. were prepared from 10 p.p.m. U and Ce solutions (CPAchem).

Total chemical analyses of the solids (Ce_0.01_U_0.99_)O_2_ and (Ce_0.1_U_0.9_)O_2_ were also carried out by dissolving 30–40 mg of the solid, both as precipitated and after 93 days of equilibration, in 2 *M* HNO_3_ (Suprapur, Merck). The resulting solutions from the dissolution were analyzed by ICP-MS. The total dissolution of a small amount of solid followed by solution analysis was carried out to check any composition change during the long-time measurements, together with X-ray diffraction (XRD) analysis of the solids.

All ICP-MS measurements were performed in triplicate. The detection limit of the ICP-MS instrument for U is 0.1 p.p.b. while that of Ce is 0.01 p.p.b.. The detection limit of the instrument was calculated by preparing and measuring the blank samples without U and Ce. The concentrations of the U and Ce in a series of blank samples were measured. The blank samples are identical to the test samples except for the absence of the analytes. It is followed by calculating the average of the blank samples’ measurements for U and Ce, then the standard deviation of the blank samples. Finally, the detection limit was calculated as three times the standard deviation of the blank samples. Measurement uncertainties were found to be quite insignificant (<2% relative uncertainty) for any concentrations above 0.1 p.p.b., due to the high resolution or detection limits of the ICP-MS instrument. The uncertainties were not plotted in the concentration series since they overlap considerably with the data points.

### 2.4. Scanning electron microscopy (SEM-EDX) analysis

A QUANTA 200 ESEM FEG scanning electron microscope (SEM) equipped with a Schottky field emission gun (FEG) for optimal spatial resolution was used to analyze the solid samples. The microscope is also equipped with an Oxford Inca Energy Dispersive X-ray (EDX) system for chemical analysis of the solid samples. The instrument was operated at high vacuum mode (HV) and an operating voltage of 30 kV. Both solid samples of (Ce_0.01_U_0.99_)O_2_ and (Ce_0.10_U_0.90_)O_2_ were analyzed before and after different equilibration periods of 21 and 90 days.

The solids were washed with 10 m*M* Na_2_S_2_O_4_ and degassed ultra-pure water to remove excess chloride ions. The washed samples were allowed to dry in an Ar atmospheric chamber glove box. The dried solids were deposited onto carbon tape.

The solids were also analyzed to determine the microstructure, the elemental composition and to also determine whether both U and Ce were homogeneously distributed in the solids. The EDX spectra for each solid particle were collected at different locations to assure uniformity within the sample and were also examined to determine whether any distinctly different phases within the solid particles occurred.

### 2.5. Powder XRD

X-ray diffraction was performed using a BRUKER D2 PHASER instrument (monochromatic Cu *K*_α_ lines, λ_1_ = 1.54184 Å) with radiation source of 2Θ range 20–90° and a LYNXEYE detector. The operating voltage and current used were 30 kV and 10 mA, respectively. *Diffrac.Topas* (V6.0) software provided by Bruker, in addition to the open access *JEdit* software, were used to determine the phase and crystal structure of the solids. The instrument was stationed or kept in the glove box with a partial pressure of O_2_ of ≤1 p.p.m. to prevent oxidation of the samples during measurements. The dried solids were analyzed before and after equilibration for phase identification and crystal structure.

About 1 g of both Ce_0.1_U_0.9_ and Ce_0.01_U_0.99_ solids were placed in alumina crucibles and heated in a furnace under an Ar + 5% H_2_ gas flow of 1 L min^−1^ at a ramp rate of 10°C min^−1^ up to 900°C, and maintained for 1 h at this temperature and then cooled down at the same rate. The solids thus obtained were also analyzed by XRD on the same instrument.

### 2.6. Surface analysis by X-ray photoelectron spectroscopy (XPS)

XPS was used to determine the oxidation states of U and Ce in the solids after equilibration. The surface measurements of the solids were carried out in a PHI5000 VersaProbe III Scanning XPS Microprobe. This system was equipped with a monochromatic aluminium (Al) X-ray source (photon energy = 1486.6 eV) with a tunable beam size between 9 µm and 300 µm in diameter. The operating beam size was set at 100 µm and the corresponding energy resolution of a core level spectrum is 0.685 eV, with reference to the full width at half-maximum (FWHM) of an Ag 3*d*_5/2_ peak measured from an ion-sputter-cleaned silver foil. The binding energy scale was calibrated in accordance with ISO 15472:2010, in which the core level of gold (Au 4*f*_7/2_), silver (Ag 3*d*_5/2_) and copper (Cu 2*p*_3/2_) were aligned at 83.96 eV, 368.21 eV and 932.62 eV, respectively. While the conductivity of the powdered samples was uncertain, dual charge neutralization was run using both argon ion gun (*i.e.* positive) and electron neutralizer (*i.e.* negative) to compensate the photoelectron loss during the measurements. Spectral measurements were divided into two steps. Survey scans were conducted with a scanning energy range between 0 and 1350 eV and step size at 1.0 eV to evaluate the composition of the samples, and then narrowed scans in the selected energy regions were scanned to evaluate the chemical states of the elements of interest with step size of 0.1 eV instead. *ULVAC-PHI MultiPak* software (Version 9.7.0.1) was used to conduct data analysis. A Shirley background was used for the background subtraction. For chemical state analysis, C 1*s* was aligned at 284.8 eV with reference to the adventitious carbon.

As the preparation chamber and XPS were located in different buildings on the campus, sample transfer from the preparation site to an XPS sample holder was conducted inside a glove box. The powdered samples were first placed onto an XPS sample holder, and then sealed into a vacuum transfer vessel inside the glove box before taken out for transportation. The transfer vessel atmosphere is thus the same as the glovebox – Ar with *p*_O2_ < 1 p.p.m. This transfer vessel, in fact, adapts to the entry port of the XPS introduction chamber. By doing this, contamination from air and moisture to the sample surfaces was reduced to a minimal level.

### 2.7. X-ray absorption spectroscopy (XAS) analysis

XAS was used to determine the oxidation state and structure around cerium and uranium in solid solutions of the mixed oxides formed. The main goal is to characterize the solid solutions after equilibration and to determine the valence of Ce and U by XAS. XAS experiments were run in transmission mode for the following samples: two solid solutions of cerium (III)–uranium (IV) oxide containing 1 and 10% cerium at both edges, Ce *L*_3_-edge (5723 eV) and U *L*_3_-edge (17166 eV), as well as pure cerium (III), cerium (IV) and uranium (IV) oxides were analyzed. Each sample contained ∼20 mg of solids, mixed with boron nitride, and covered with Kapton tape.

Uranium and cerium *L*_3_-edge X-ray absorption data were collected in transmission mode at ambient temperature at the Balder beamline, MAX IV Laboratory, Lund University, Sweden (Klementiev *et al.*, 2016 ▸), operated at 3 GeV and a current of 500 mA run in top-up mode. A Si[111] double-crystal monochromator was used, and mirrors to reject higher harmonics. The solid samples were kept in cells made of 1 mm aluminium frames with Kapton tape as windows. The X-ray absorption spectra were energy calibrated using a chromium metal foil, 5989.0 eV (Thompson *et al.*, 2009 ▸) for the cerium measurements, and an yttrium metal foil, 17038.0 eV (Thompson *et al.*, 2009 ▸) for the uranium measurements. The experimental data were treated by using standard procedures for pre-edge subtraction and spline removal and Fourier transformation by means of the program package *EXAFS­PAK* (George & Pickering, 1993 ▸). *Ab initio* calculated EXAFS parameters, generated by the program *FEFF* v.7.0 (Zabinsky *et al.*, 1995 ▸), were used in the curve-fitting procedure.

## 3. Results

### 3.1. Spectrophotometric analysis of U stock solution

**Figure 1:**
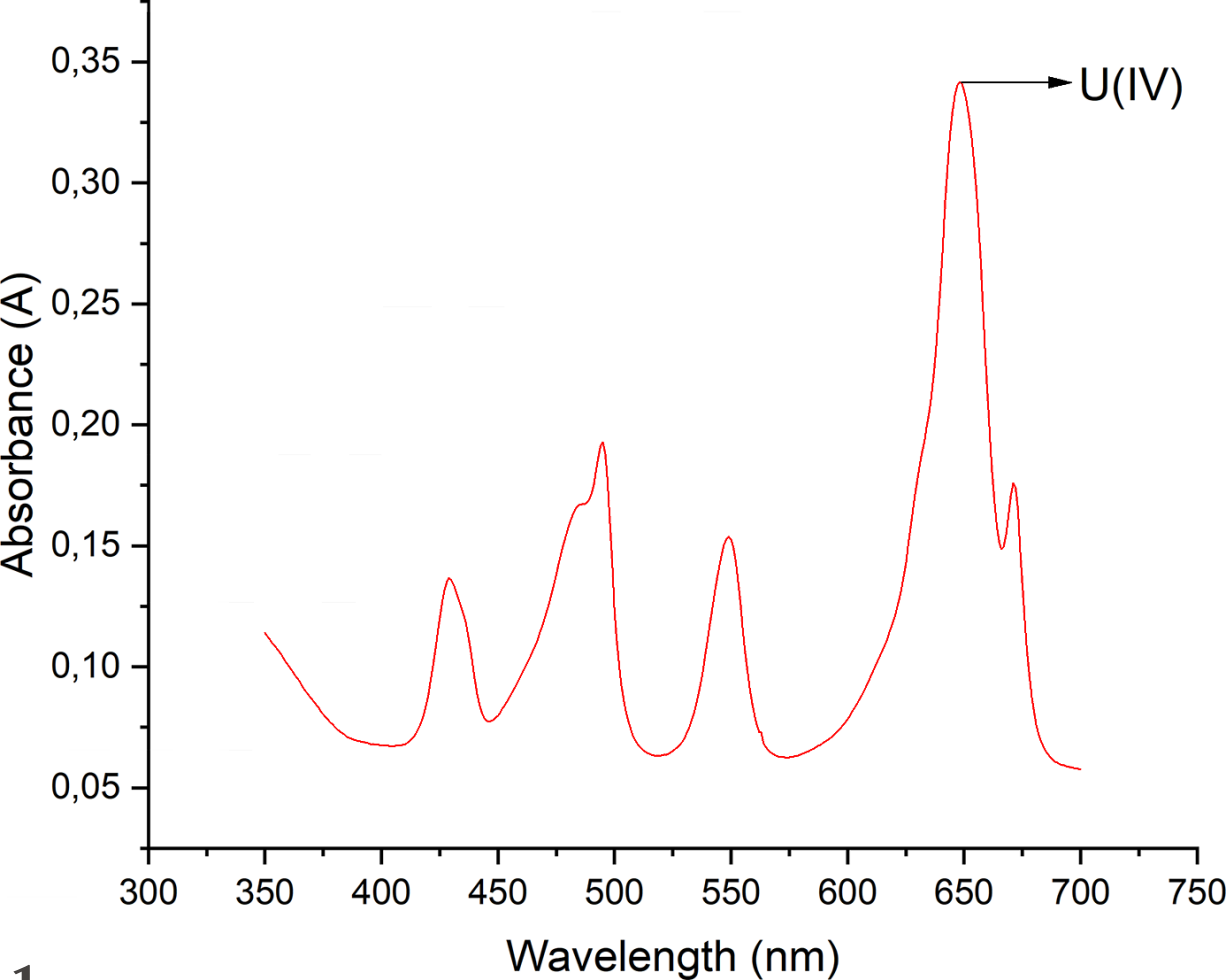
UV-visible spectrophotometric analysis of U stock solution.

The UV-vis spectrophotometric analysis of the prepared stock solution (see Fig. 1 ▸) showed the presence of a U (IV) characteristic peak at 648 nm and the absence of any other major peak for other oxidation states such as U (VI) at 414 nm. Details are given in the supporting information.

### 3.2. Kinetics of solubility equilibria

**Figure 2:**
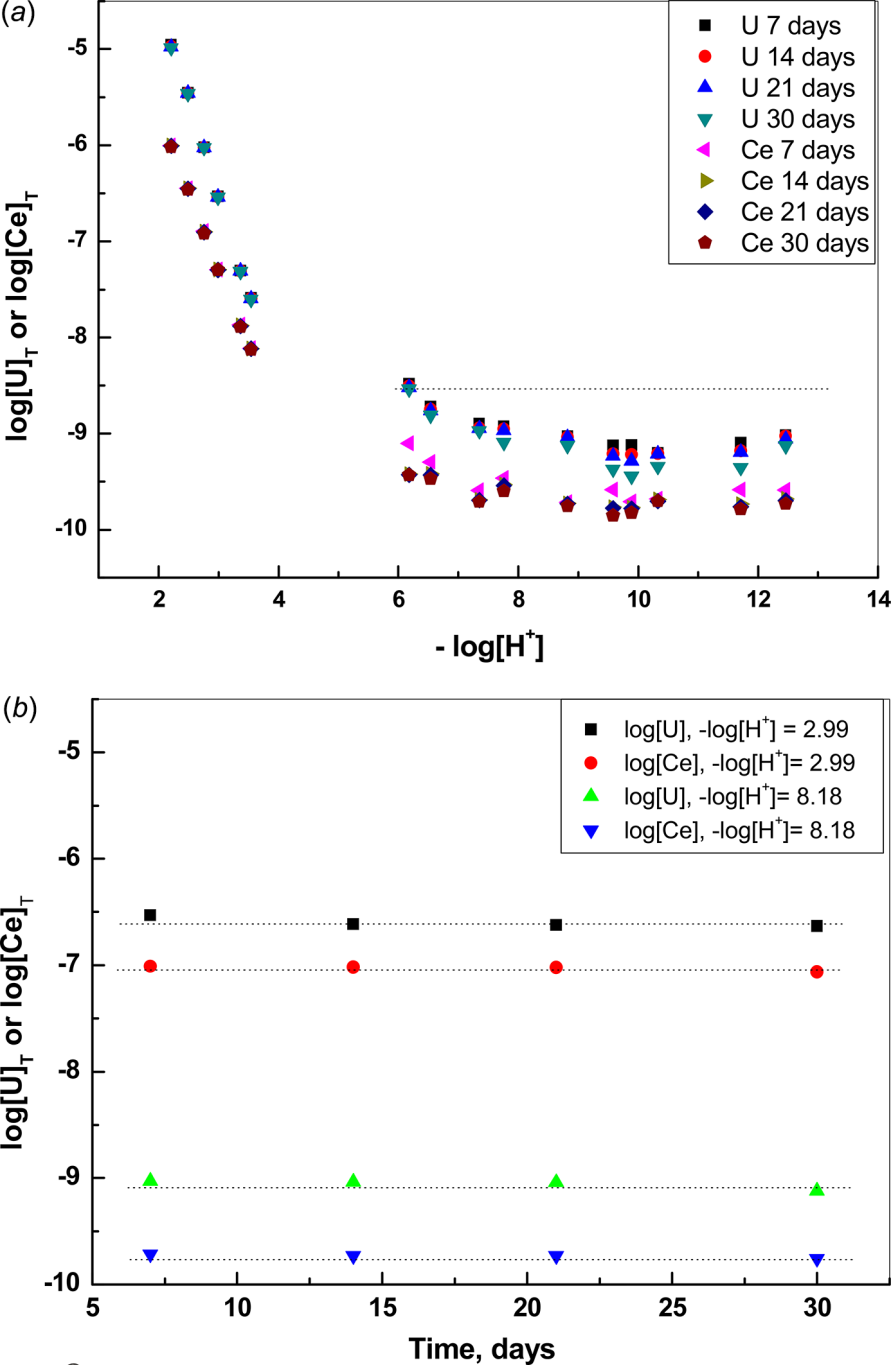
(*a*) Evolution of U and Ce total concentrations during 30 days equilibration for all pH values investigated for the solid with 1% Ce. (*b*) Evolution of U and Ce concentrations during 30 days equilibration for two randomly selected samples at −log[H^+^] values of 2.99 and 8.18. The dotted lines are horizontal and centered at the value of the last point.

As can be seen in Fig. 2 ▸(*a*), the data for the total concentration of U and Ce in contact with U_0.99_Ce_0.01_O_2_ are quite similar after 7, 14, 21 and 30 days with only a very slight decrease in concentration with time due to the aging of the amorphous solid. This slight decrease in concentration is the expected behavior of amorphous solids and it would certainly become larger for longer equilibration times. In Fig. 2 ▸(*b*) the evolution in time of U and Ce concentrations in contact with UO_2_ containing 1% Ce for two selected pH values shows that only minor changes occur after the first 7 days. A similar behavior was observed in other tests with amorphous UO_2_ based coprecipitates: Bruno & Sandino (1988 ▸) report steady state U concentrations after 50 h in contact with the solid, and Rai *et al.* (2004 ▸) and references therein report that equilibrium in these systems is reached in about 3 days. Therefore, our data in conjunction with literature data on amorphous UO_2_ based coprecipitates can be used to conclude that equilibrium was most certainly reached in the present study. These equilibrium conditions correspond to equilibrium of both components of the freshly precipitated solids with the solution; at longer equilibration times the aged coprecipitates are expected to become more crystalline, which will result in lower solubilities.

### 3.3. Characterization of solid phases

#### 3.3.1. Total chemical analysis

The total molar ratios of U and Ce were determined for solid samples before and after equilibration. The solids (a few mg) were dissolved completely in 2 *M* HNO_3_ and the resulting solution was analyzed by ICP-MS for U and Ce. The composition of the solid phase after 93 days at equilibrium with a solution at pH 8.2 was quite similar to the solid before equilibration, as shown in Table 1 ▸. The analysis was also carried out for 30 days with similar results to the 93 days analysis. The longer equilibration period was chosen in analogy to Rai *et al.* (2004 ▸), who reported solid analysis for a longer equilibration period of 238 days against the solubility data reported for 38 days.

#### 3.3.2. SEM-EDX results

**Figure 3:**
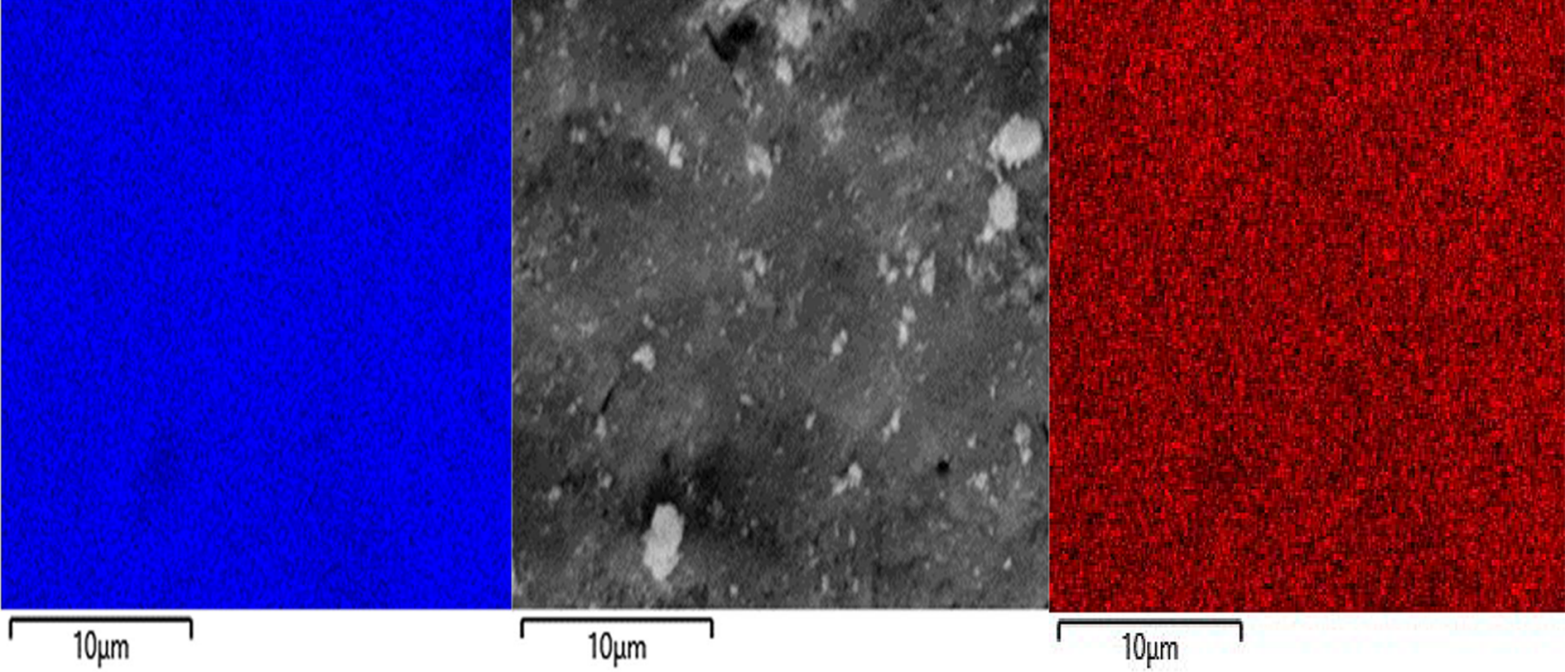
SEM micrograph of (Ce_0.1_U_0.9_)O_2±*x*_ solid (center) and SEM-EDX U mapping (left) and Ce mapping (right) of the same area. Analysis made before equilibration.

The U–Ce solid samples before and after equilibration were analyzed by SEM-EDX in the back-scattered electron mode. The micrographs obtained show that the solids are uniform in appearance and no contrasting phases can be observed, ruling out higher concentrations of U or Ce in one area. The EDX analyses of the 10% Ce sample (see Figs. 3 ▸ and 4 ▸) show that U and Ce appear homogeneously distributed in the solid sample. An initial scan of the powder in the SEM sample holder was carried out, followed by multiple scans of selected areas or parts which looked interesting due to formation of agglomerates or other features. Homogeneity was assumed since in all cases when we performed an EDX analysis it showed both U and Ce homogeneously distributed, as shown, for example, in Figs. 3 ▸ and 5 ▸.

**Figure 5:**
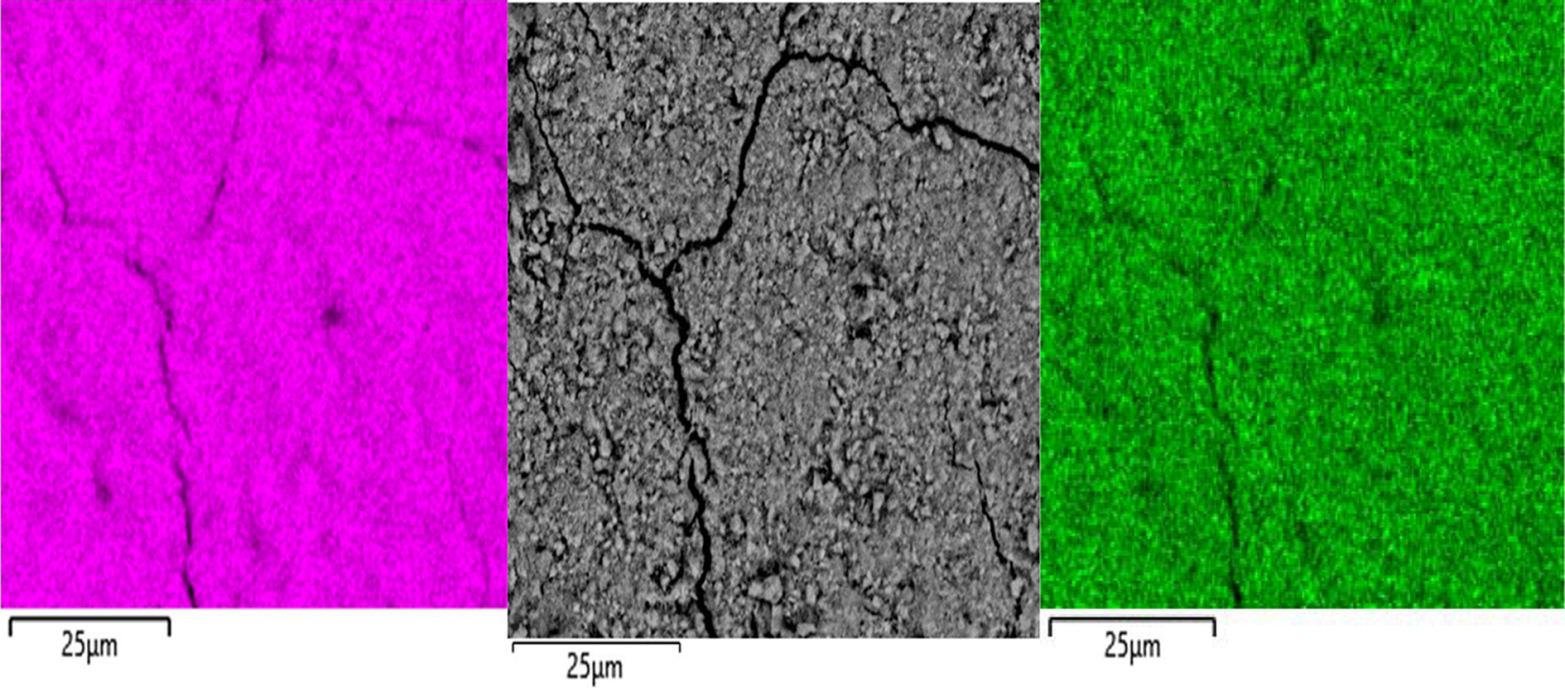
SEM micrograph of (Ce_0.1_U_0.9_)O_2±*x*_ solid (center) and SEM-EDX U mapping (left) and Ce mapping (right) of the same area. Analysis made after equilibration.

The 1% Ce sample was below the detection limit of the EDX analysis. It can be concluded that solid samples contain a single phase rather than a mixture of two different solid phases and from the homogeneous distribution of U and Ce in the corresponding EDX mappings it follows that the solid samples are most likely a solid solution. No other phases could be detected in the solids after equilibration and the same homogeneous distribution of U and Ce was observed in the solid after equilibration (see Fig. 5 ▸).

#### 3.3.3. XRD results

**Figure 6:**
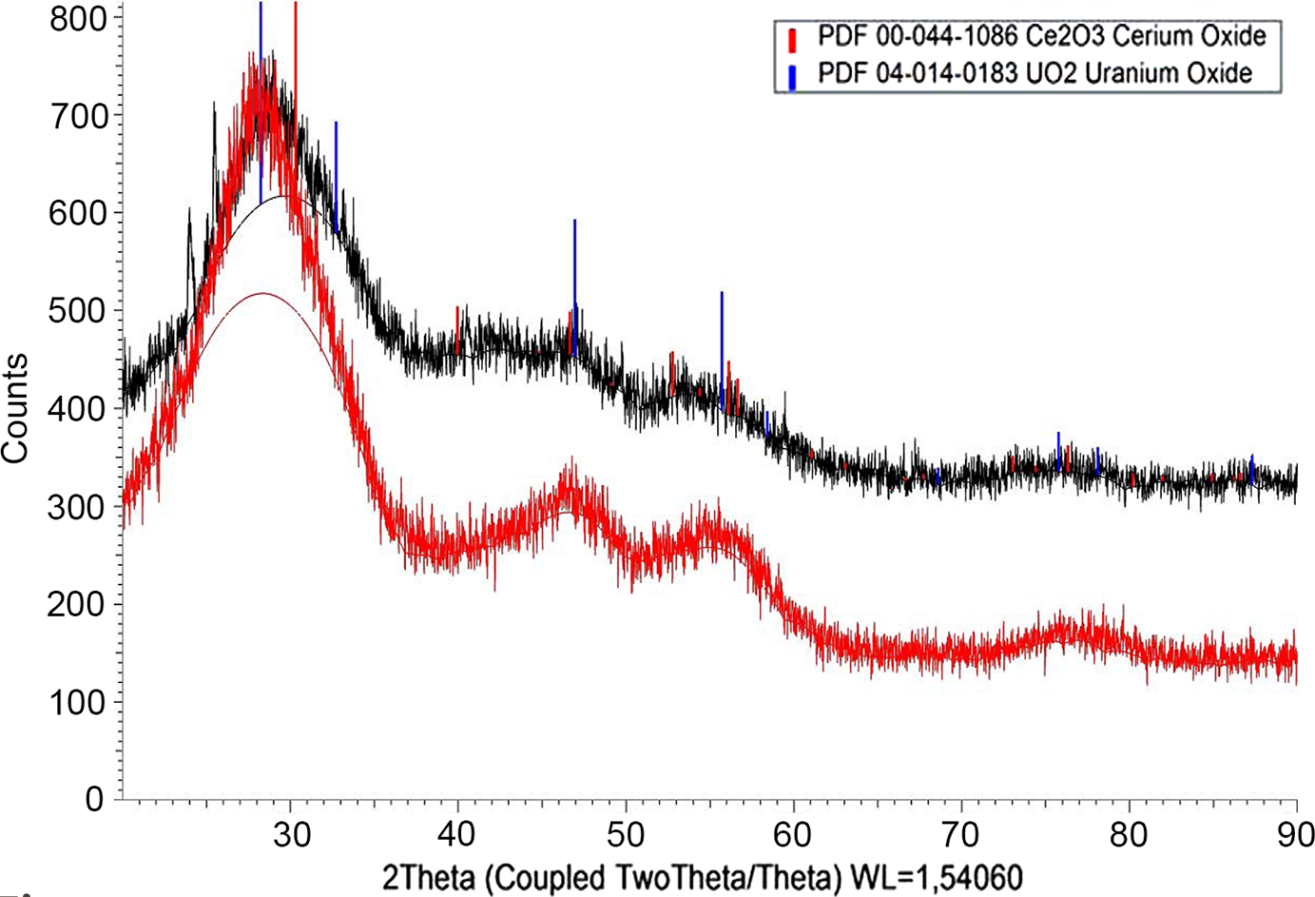
XRD spectra of the Ce_0.10_U_0.90_ solid before (upper) and after (lower) equilibration.

The diffraction peaks of the solids before and after equilibration were broad and similar in appearance (see Fig. 6 ▸). The representative solid samples show broad peaks and not well defined peaks. The solid precipitates’ peaks indicate that the solids are mainly amorphous, and the presence of micro-crystallinity cannot be ruled out. It can be concluded that the solubility controlling solids are amorphous. The International Centre for Diffraction Data (ICDD) database was used for indexing, specifically the PDF 2 and PDF 4 databases.

**Figure 7:**
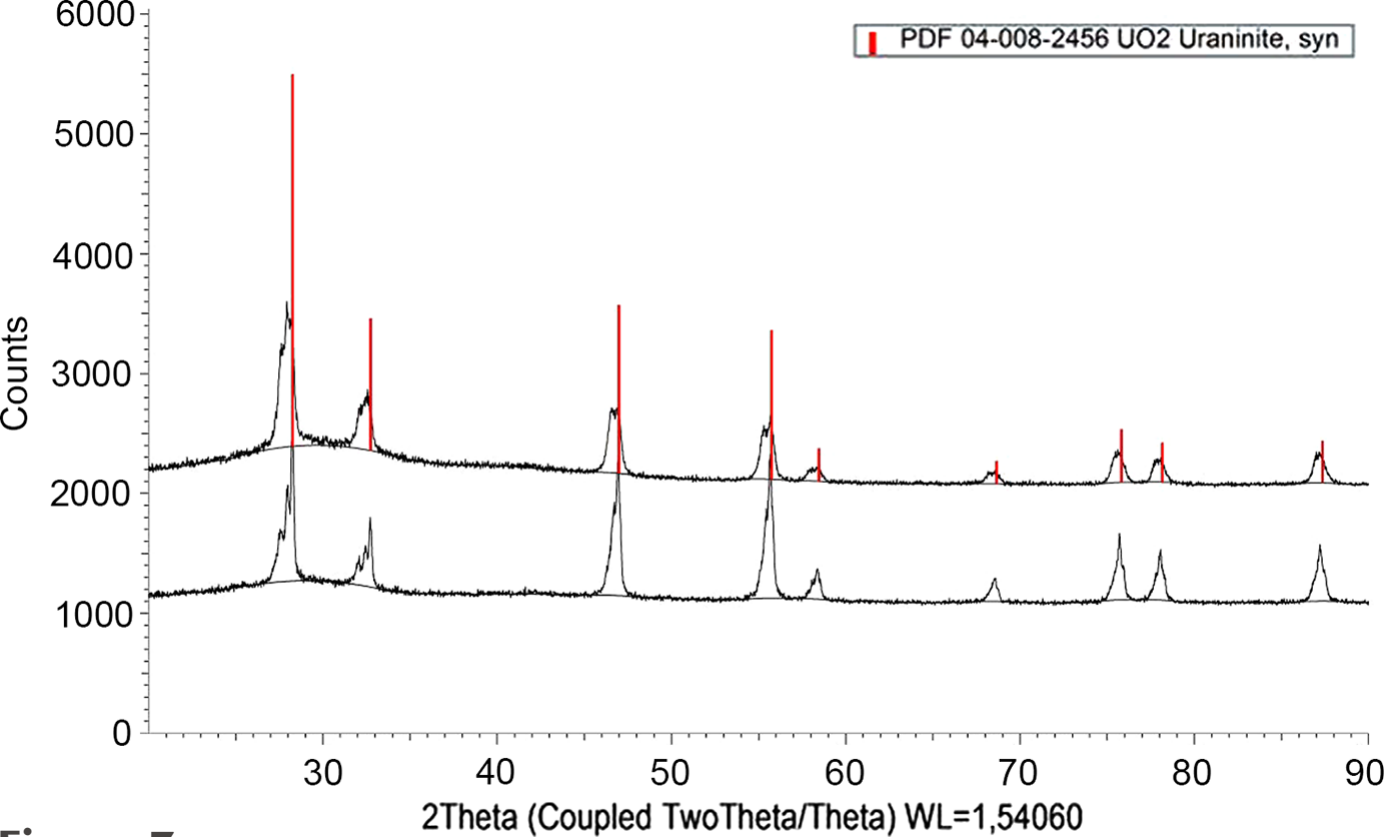
U–Ce equilibrated solid heated at 900°C under reducing conditions. The upper spectrum is for Ce_0.10_U_0.90_ while the lower spectrum is for Ce_0.01_U_0.99_.

The samples sintered at 900°C under reducing atmosphere had well defined crystalline narrow peaks for both samples, as shown in Fig. 7 ▸.

The lattice parameters were refined using the *GSAS-II* software (Toby and Von Dreele, 2013 ▸). As discussed by Kleykamp (1993 ▸), the lattice parameter of the solid solution contracts compared with pure UO_2_ with the increase of Ce content for stoichiometric solids. In the case of cerium, the relationship for the lattice parameter of the solid solution (*a*) is related to the mole fraction of Ce (*x*) and the lattice parameter of UO_2.00_ by the relationship (Mclver, 1966 ▸)
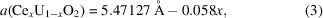
where the lattice parameter of UO_2.00_ is 5.47127 Å (Leinders *et al.*, 2015 ▸).

The refined lattice parameters for our coprecipitates are shown in Table 2 ▸. They are in good agreement with the expected trend for the two solid solutions investigated and show a linear decrease of the cell parameter with Ce content, however with a slightly lower slope. This may be an indication of near stoichiometric solid solutions formed in our case; however, the ratio O/*M* was not investigated in this study.

As the Ce content in the solid solution increases, the lattice parameter of the solid solution contracts when compared with pure UO_2_. These changes in the lattice parameter are usually attributed to changes in the UO_2_ stoichiometry or to the presence of oxygen vacancies. The substitution of U with other cations of lower charge such as Ce^3+^ implies either the formation of oxygen vacancies or an increase of the U oxidation state (Prieur *et al.*, 2018 ▸). Both mechanisms are equivalent and depend mostly on the experimental method used for the structural investigation of the oxide: either a spectroscopic method for the electronic structure of the cations (*e.g.* XAS) or diffraction methods for investigating the oxygen lattice (*e.g.* neutron diffraction).

#### 3.3.4. XPS results

**Figure 8:**
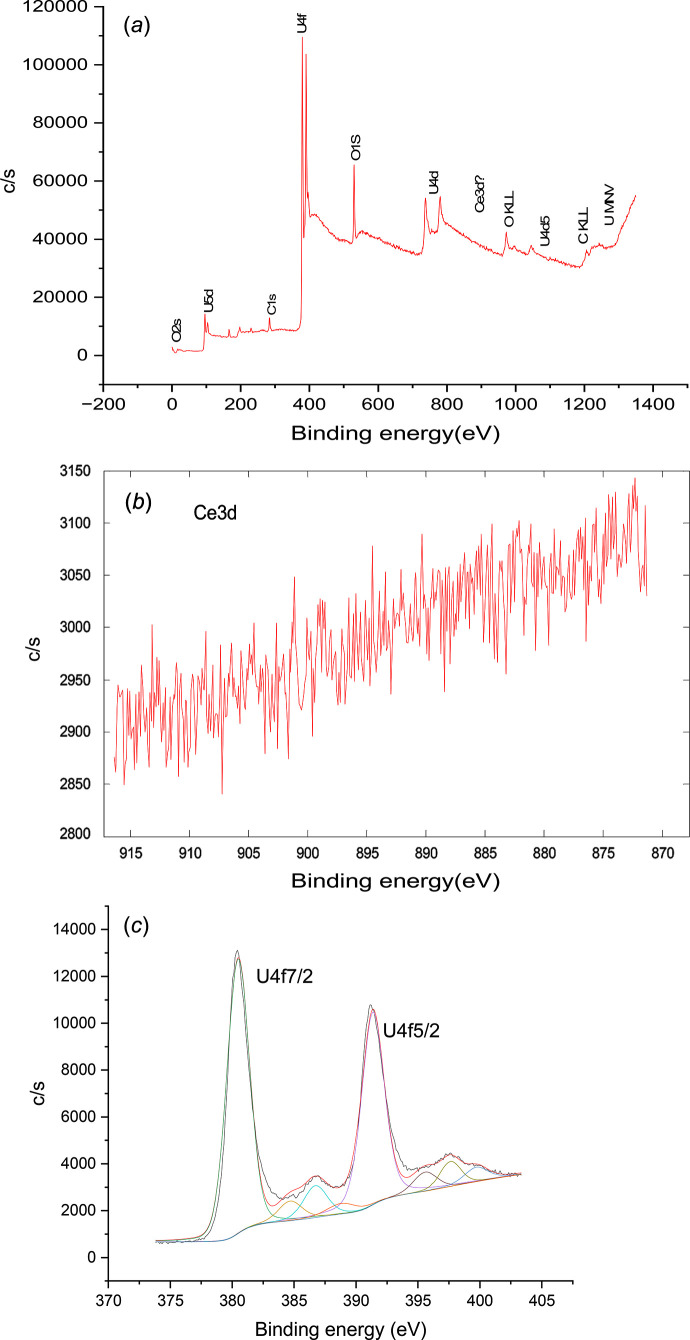
Survey XPS spectrum of the as-received Ce_0.01_U_0.99_ sample. (*b*) High resolution XPS scans in the Ce 3*d* region for the Ce_0.01_U_0.99_ solid. (*c*) High resolution XPS scans in the U 4*f* region for the Ce_0.01_U_0.99_ solid.

*Ce_0.01_U_0.99_ solid.* The acquired survey spectrum of U_0.99_Ce_0.01_ from the as-received condition in Fig. 8 ▸(*a*) shows that surface contaminants including carbon and sulfur from di­thio­nite are determined besides the expected elements. The oxygen content is mainly contributed from the native oxide of the sample. Cerium content, in fact, is below the system detection limit (*i.e.* 1.0 at%), and therefore does not show any feature throughout the whole range. Even when zooming into the Ce 3*d* region in the narrow scan [Fig. 8 ▸(*b*)], no significant spectral line is detected either. Meanwhile, a high resolution scan in the U 4*f* region [Fig. 8 ▸(*c*)] shows the presence of the U (IV) state by the presence of a U 4*f*_7/2_ peak at 380.5 eV, a spin–orbit splitting of 10.90 eV with the U4 *f*_5/2_ peak, and a clear indication of the satellite peaks from each doublet (Hansson *et al.*, 2021 ▸). The absence of U 4*f*_7/2_ at 381.0 eV or above (Hansson *et al.*, 2021 ▸; Ilton & Bagus, 2011 ▸) indicates that the U (VI) state is absent, or at least below the XPS detection limit. With that, the presence of uranium (IV) oxide in the Ce_0.01_U_0.99_ sample is confirmed.

**Figure 9:**
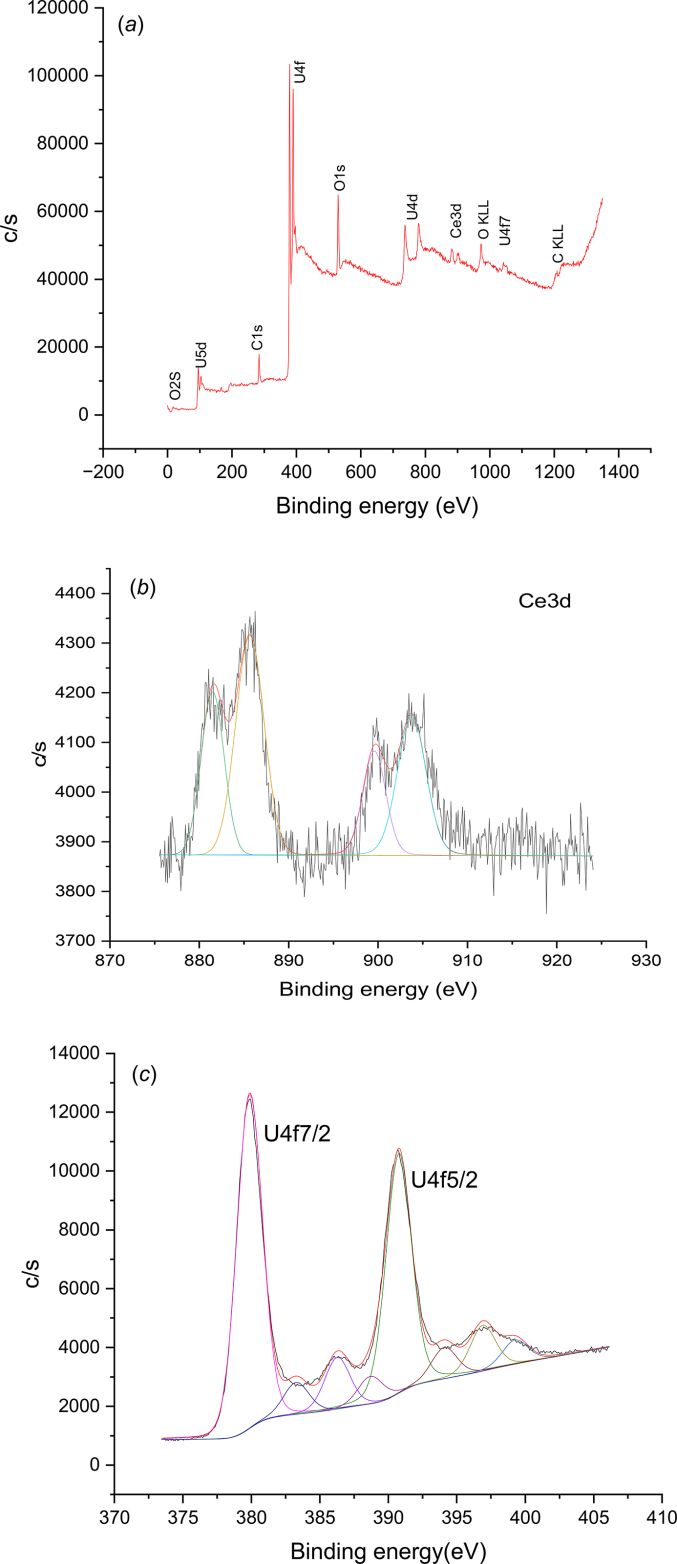
(*a*). Survey XPS spectrum of the as-received Ce_0.10_U_0.90_ sample. (*b*) High resolution XPS scans in the Ce 3*d* for the Ce_0.10_U_0.90_ solid. (*c*). High resolution XPS scans in the U 4*f* region for the Ce_0.10_U_0.90_ solid.

*Ce_0.10_U_0.90_ solid.* The survey spectrum in Fig. 9 ▸(*a*) shows the composition of the Ce_0.10_U_0.90_ sample at the as-received condition. Like Ce_0.01_U_0.99_ solid, surface contaminants including carbon and sulfur are determined besides the expected elements. The presence of oxygen is probably contributed by the native oxide. By deducting their contribution, the U:Ce ratio comes close to 9:1. A high resolution scan in the Ce 3*d* region [Fig. 9 ▸(*b*)] shows two pairs of spin–orbit doublets of Ce 3*d*_5/2_ and Ce 3*d*_3/2_, corresponding to the characteristic features of the Ce (III) state (Paparazzo, 2018 ▸). A high resolution scan in the U 4*f* region [Fig. 9 ▸(*c*)] shows the presence of the U (IV) state, by a U 4*f*_7/2_ peak at 379.5 eV, a spin–orbit splitting with U 4*f*_5/2_ at 10.90 eV, and a clear indication of the satellite peaks from each doublet (Hansson *et al.*, 2021 ▸). With that, the presence of uranium (IV) oxide (UO_2_) and cerium (III) oxide (Ce_2_O_3_) in a ratio of 9:1 is determined in the Ce_0.10_U_0.90_ sample.

#### 3.3.5. XAS results

**Figure 10:**
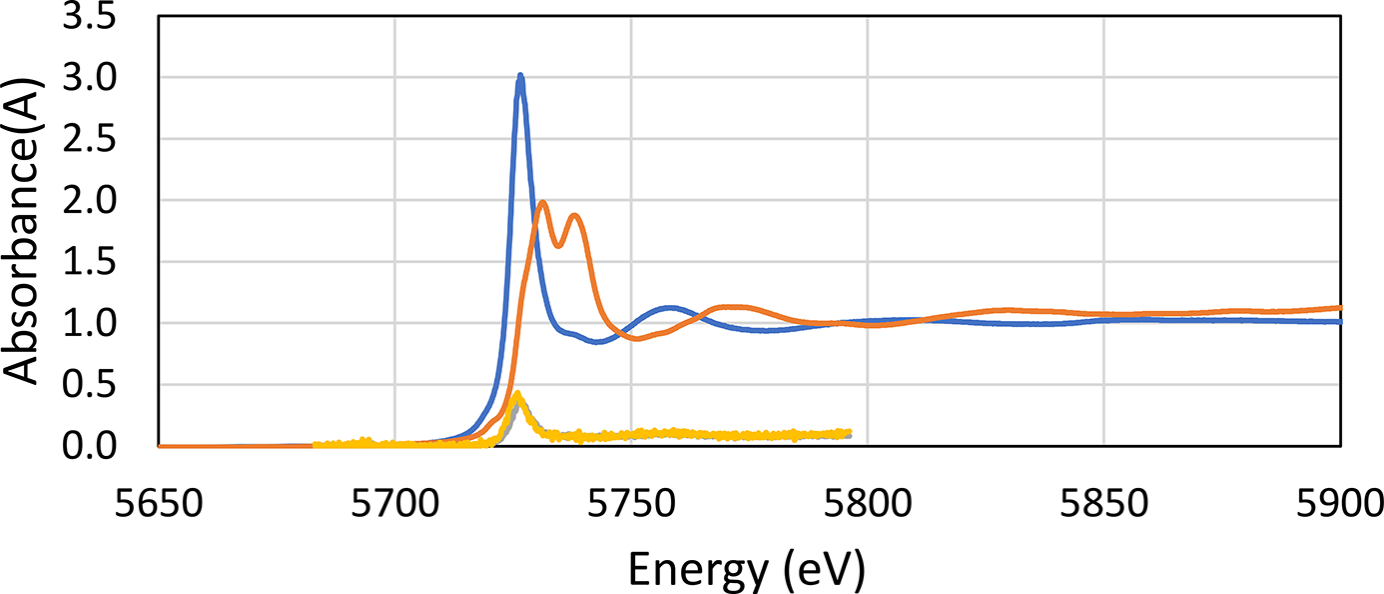
Normalized raw XANES spectra of cerium (III) oxide, Ce_2_O_3_ (light blue line) and cerium (IV) oxide, CeO_2_ (brown line). Raw data, not normalized, of U_0.90_Ce_0.10_ at two different spots (yellow and gray lines) show that cerium is present as cerium (III) in the studied samples.

**Figure 11:**
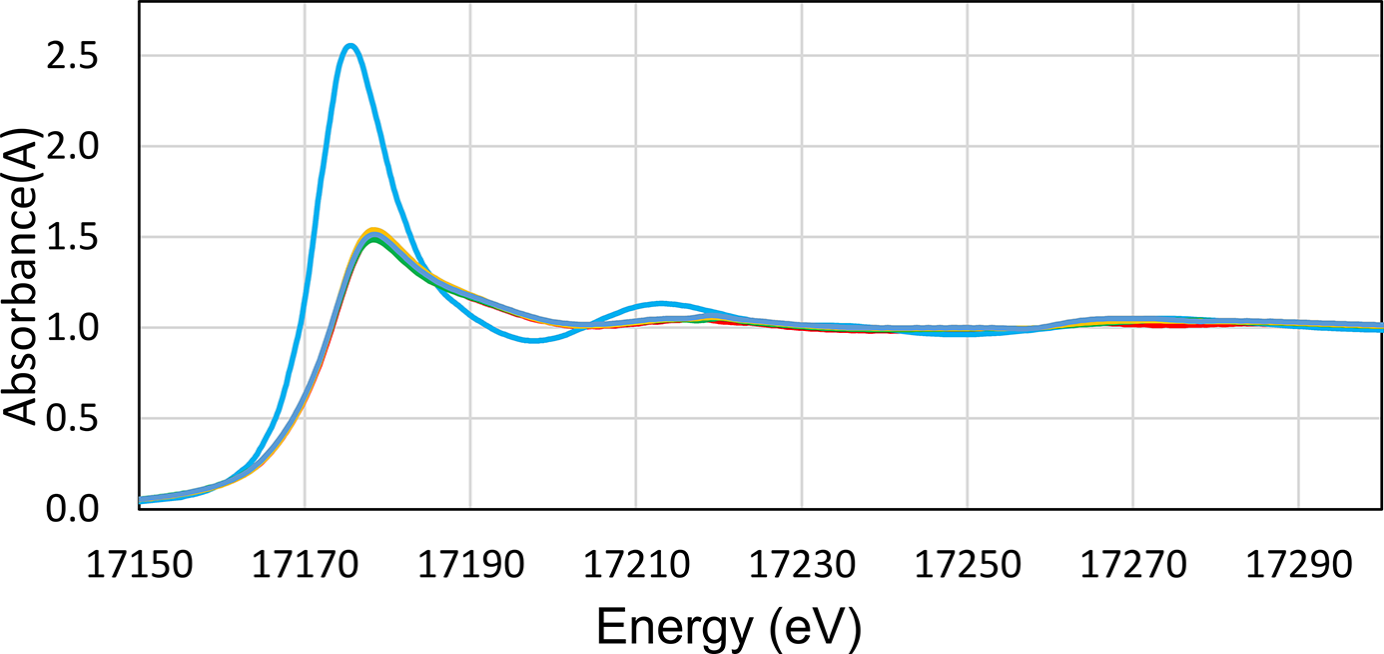
Normalized raw XANES spectra of uranium (IV) oxide, UO_2_ (light blue line), and the studied uranium samples, ‘sample UO_2__pure_1’ (red line), ‘sample UO_2__pure_2’ (green line), ‘sample U_0.99_Ce_0.01_’ (purple line) and ‘sample U_0.90_Ce_0.10_’ (yellow line). Note that samples ‘UO_2__pure_1’, ‘UO_2__pure_2’, ‘U_0.99_Ce _0.01_’ and ‘U_0.90_Ce_0.10_’ all consist of uranium (VI) oxide.

**Figure 12:**
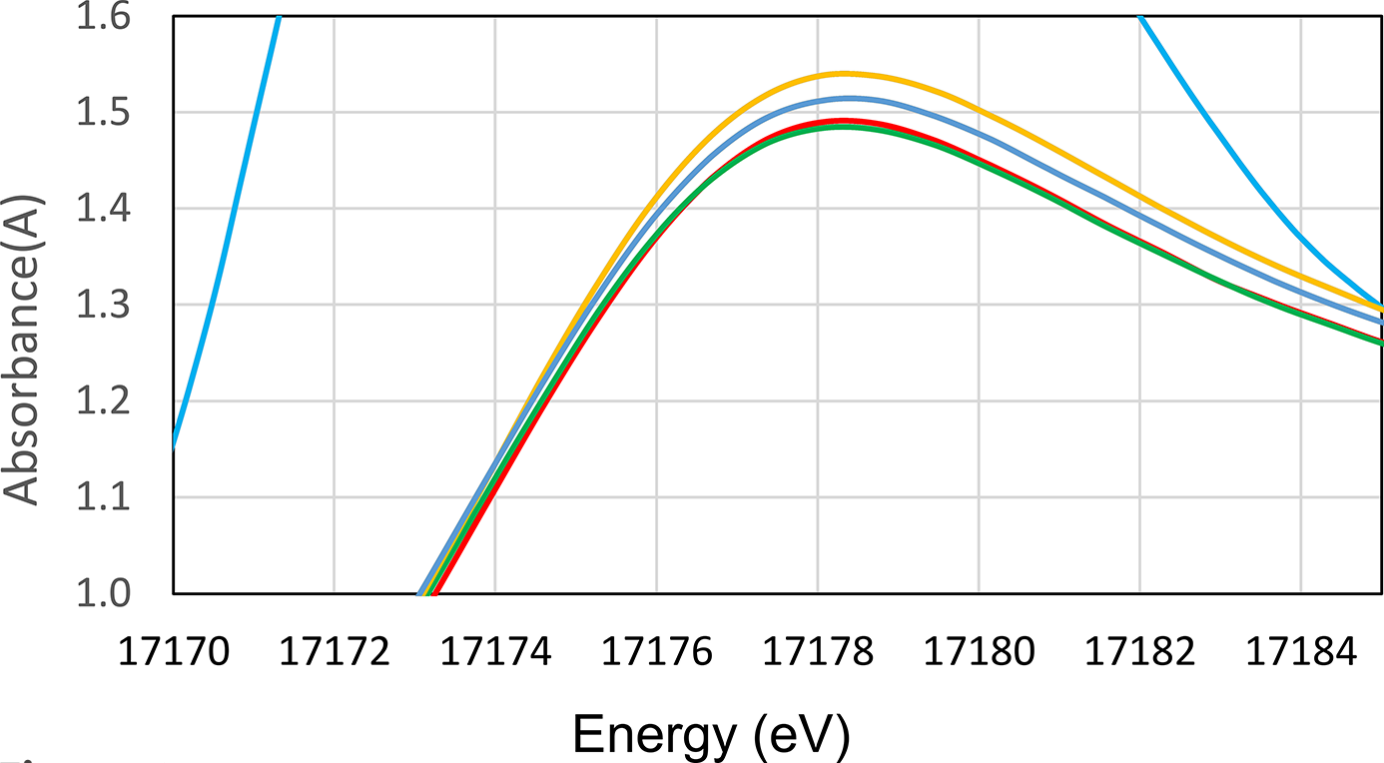
Close up of Fig. 11 ▸ at the white line peak region

*Analysis of XANES data.* Cerium is present as cerium (III) in the U–Ce solid samples as confirmed with XANES (see Fig. 10 ▸). A comparison of the XANES of crystalline uranium (IV) oxide, UO_2_, collected in 2013, and the uranium oxide samples under study is given in Fig. 11 ▸. The collected XANES data obtained on the Balder beamline for the studied solid samples shows that that the uranium consists of uranium (VI) oxide, UO_3_. The XANES spectra are very different, and in perfect agreement with previous XANES studies of uranium (IV) and uranium (VI) oxide (Leinders *et al.*, 2020 ▸; Allen *et al.*, 1996 ▸). There is a very small difference in the XANES spectra of pure uranium (VI) oxide with those containing 1 and 10% cerium (III) oxide, which have increasing white line intensity with increasing cerium content (see Fig. 12 ▸).

**Figure 13:**
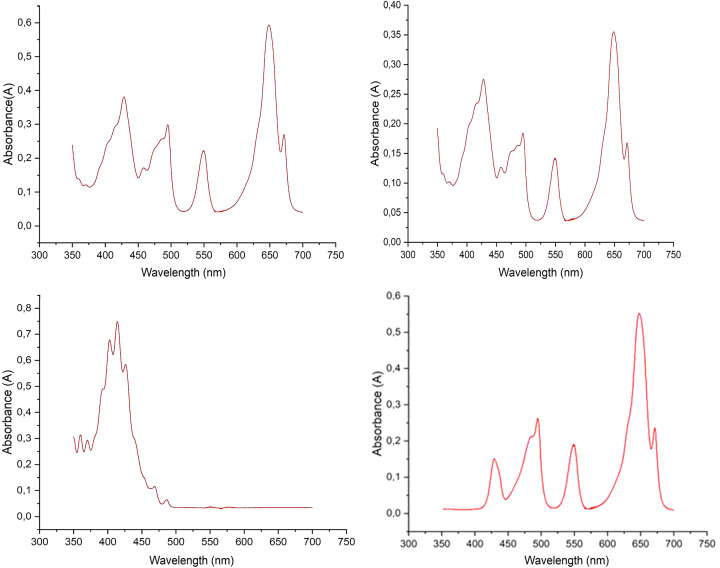
UV-vis analysis of the MAX IV laboratory samples after dissolution in HClO_4_ (top; left Ce_0.01_U_0.99_, right Ce_0.10_U_0.90_) compared with spectra of pure U (VI) and U (IV) in the same solvent (bottom).

*Analysis of EXAFS data.* With the information from the XANES region, the EXAFS data were refined. The refined structure parameters are typical for uranyl (VI) complexes with two strong U=O bonds at 1.785 Å, and linear multiple scattering within the O=U=O entity. There are about four U—O bonds at 2.32 Å, and four U⋯U distances at ∼3.45 and 4.17 Å (see Table 3 ▸ for details). These distances strongly indicate that the uranium sample consists of γ-UO_3_. No U—Ce distance was observed from the EXAFS analysis. After receiving these results, we decided to dissolve completely a certain amount of the solid sample used for XAS and XANES analysis in perchloric acid and analyze by UV-vis spectroscopy the oxidation state of uranium. The results for the two samples analyzed at MAX IV Laboratory and those for pure U (IV) and U (VI) solutions are presented in Fig. 13 ▸ and show that most of the uranium in the sample is U (IV).

We will investigate further the cause of the oxidation of the MAX IV laboratory samples, but this does not affect the conclusions of the paper discussed in continuation.

### 3.4. Solution concentrations and solubility of the coprecipitates

**Figure 14:**
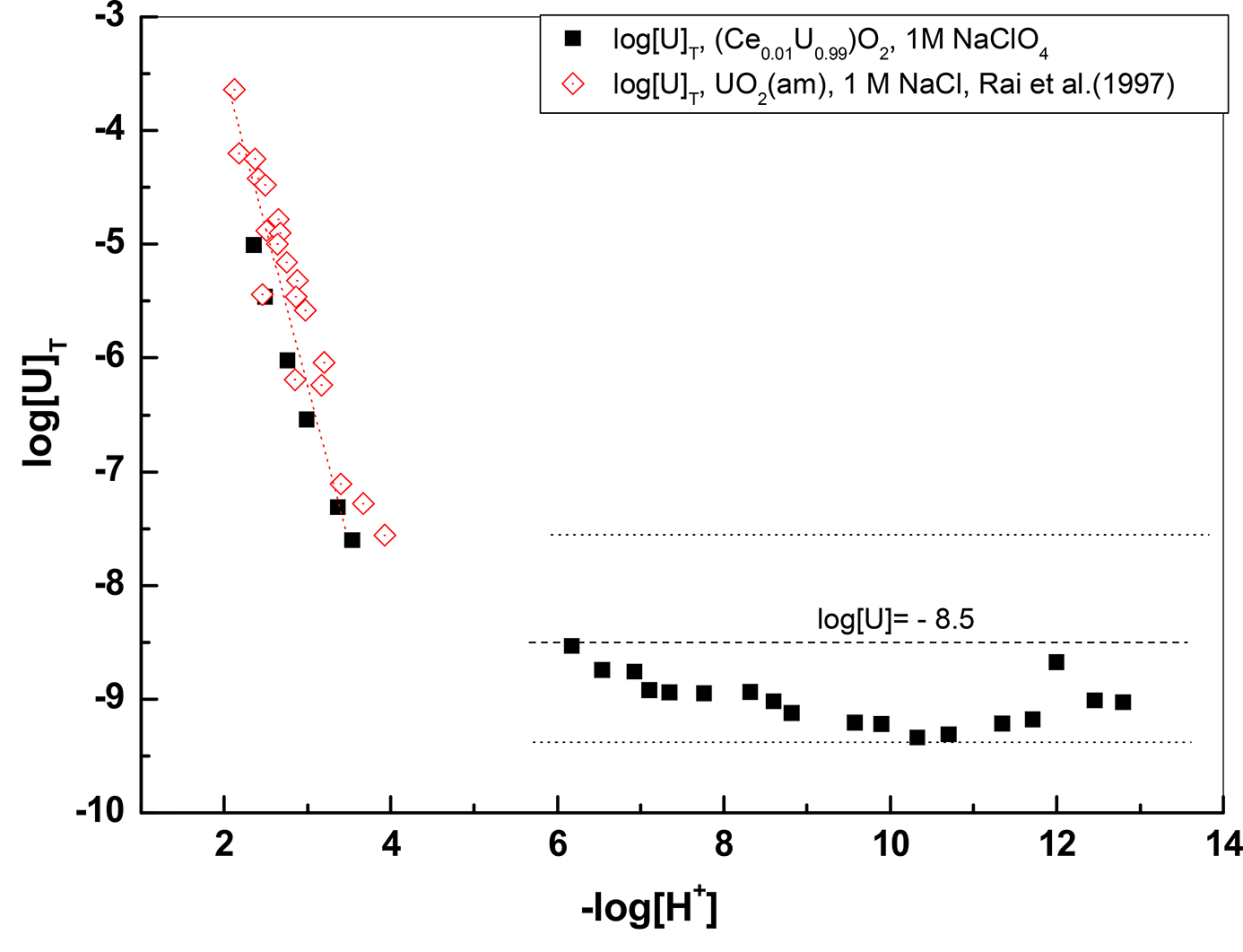
Concentrations of U in equilibrium with the Ce_0.01_U_0.99_ solid solution at 30 days. The dotted line with slope −3 is from Fig. 5 of Rai *et al.* (1997 ▸), while the horizontal dotted lines indicate log[U] = −8.5 ± 1. The data of Rai *et al.* (1997 ▸) are for equilibration times 8–420 days.

The behavior of U in equilibrium with the solid containing only 0.01 mol fraction of Ce is expected to be quite similar to that of amorphous UO_2_(s). In Fig. 14 ▸ we compare our U concentration data at 30 days with the U data from solubility measurements of UO_2_(am) in 1 *M* NaCl (Rai *et al.*, 1997 ▸). In the recent Nuclear Energy Agency Thermochemical Database (NEA-TDB) volume on actinides (Grenthe *et al.*, 2020 ▸), the study by Fujiwara *et al.* (2003 ▸) on the solubility of UO_2_(am) is reported to contain data in 1 *M* NaClO_4_, but in fact these are data obtained in 1 *M* NaCl obtained in their previous study (Fujiwara *et al.*, 2002 ▸) and were not included in the comparison because they are slightly higher than those obtained by Rai *et al.* (1997 ▸). As seen from the figure, the U data are quite like those of Rai *et al.* (1997 ▸) in the –log[H^+^] range 2–4, where a decrease of U concentrations by approximately three orders of magnitude for each unit −log[H^+^] increase is observed. The dominating hydrolysis complex for U (IV) at this pH interval is expected to be the first hydrolysis complex U(OH)^3+^, as shown by several studies on UO_2_(am) solubility. Thus, the relevant equilibrium reaction for UO_2_(am) with solution in this pH interval is
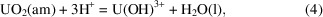

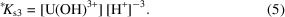
The slightly lower U concentrations observed for the co­precipitate in the acidic range cannot be due to the presence of Ce because similar U concentrations are observed in the case of the Ce_0.1_U_0.9_ solid (Fig. 16).

The U concentrations for −log[H^+^] higher than 4 agree well with the lower limit of the selected solubility for UO_2_(am) in this pH range (Grenthe *et al.*, 2020 ▸; log[U] = −8.5 ± 1) and with uranium concentrations in several spent fuel leaching tests at pH around 8 (Ekeroth *et al.*, 2020 ▸; Spahiu *et al.*, 2004 ▸; Puranen *et al.*, 2020 ▸). The constant 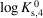 = −8.5 corresponds to the equilibrium UO_2_(am) + 2H_2_O = U(OH)_4_(aq) and has practically no ionic strength dependence, since it involves no charged ions [the Debye–Hückel term in specific ion interaction theory (SIT) is zero] and involves an uncharged complex. It has been reported with the same value of −8.5 even at 5 *M* NaCl (Çevirim-Papaioannou *et al.*, 2018 ▸). No attempt was made to check the oxidation state of U or Ce at such low concentrations, but our U data correspond to the lowest reported in the literature for such systems. This excludes any presence of U (VI), and if U is not oxidized it is reasonable to assume that also Ce is not.

The measured Ce (III) concentrations in equilibrium with the Ce_0.01_U_0.99_ coprecipitate are slightly less than an order of magnitude lower than those of U for pH higher than 6, while in the −log[H^+^] region 2–4 the decrease is higher than an order of magnitude at the lowest pH measured and becomes slightly less as the pH increases. In any case, one can exclude the presence of any trace of pure Ce(OH)_3_(s) in the solid, because it would dissolve completely in the pH interval 2–4 and give rise to much higher Ce concentrations than those measured. The measured Ce concentrations indicate also clearly that the coprecipitate does not behave as a homogeneous ideal solid solution in which case the concentrations of Ce should decrease proportionally to the mole fraction of Ce (0.01) as compared with the concentrations in equilibrium with the pure solid Ce(OH)_3_(s). According to the data of Kragten & Decnop-Weever (1978 ▸) in 1 *M* NaClO_4_, Ce(OH)_3_(s) starts to precipitate at pH around 7 and would dissolve completely at pH < 4.

For each chemical component in the system UO_2_.*x*H_2_O(s)–Ce(OH)_3_(s)–H_2_O at equilibrium the chemical potential (μ) must be the same in the solid and in the aqueous phase, μ^s^[Ce(OH)_3_] = μ^aq^[Ce(OH)_3_]. In our case we have concentration data for Ce and U in equilibrium with the mixed oxide phase for two pH regions where the aqueous species are expected to be different: −log[H^+^] of 2.2–3.6 and −log[H^+^] of 6.5–12.8.

In the low pH region 2.2–3.6 the dominating equilibrium for dissolution of Ce(OH)_3_(s) can be written
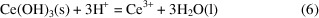
since no hydrolytic Ce (III) complexes are expected to exist at such a low pH range. The thermodynamic equilibrium constant for Ce(OH)_3_(s) in the coprecipitate and the solution can be expressed as
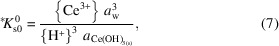
where 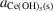 is the activity of Ce(OH)_3_(s) in the solid solution and the curved brackets denote the activities of the species in solution. From equation (7) we have for the concentration of Ce in equilibrium with the coprecipitate at 1 *M* NaClO_4_,
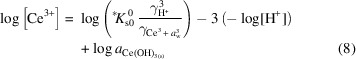
where the constant activity coefficients and the activity of water at 1 *M* NaClO_4_ are included in the conditional constant 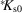.

The conditional solubility product 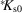 of Ce(OH)_3_(s) at 1 *M* NaClO_4_ has been determined by Kragten & Decnop-Weever (1978 ▸) as
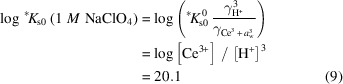
by including again the constant activity coefficients and the activity of water at 1 *M* NaClO_4_ in the conditional constant 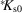. Equation (7) can be written for the coprecipitate in 1 *M* NaClO_4_ as
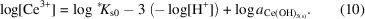


At fixed temperature, pressure and composition, *a*_Ce(OH)3(s)_ is constant if the free energy of the solid is constant. Aging of the precipitate usually lowers the free energy of the solid, but in our case this lowering was quite small for 30 days (see Fig. 2 ▸). The activity of Ce(OH)_3_(s) in the solid solution can be estimated by introducing a solid phase composition dependent constant denoted *K*_*x*_, where *x* is the mole fraction of Ce in the solid,
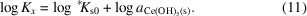
The constant *K*_*x*_ equals 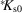 when Ce(OH)_3_(s) is in its standard state, *i.e.* pure Ce(OH)_3_(s). The constant *K*_*x*_ can be expressed in this log[H^+^] range by combining equations (10) and (11) as
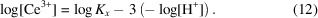
As seen from equation (12), it is possible to evaluate the constant *K*_*x*_ by fitting the log[Ce^3+^] data in the acidic −log[H^+^] range to a line of slope −3 and extrapolating to −log[H^+^] = 0. The real slope of our Ce data is much closer to 2 than to 3, but we consider the existence of hydrolysed Ce(OH)^2+^ species at such low pH impossible. This inconsistency seems to be due more to the evaluation of the solubility of a solid which cannot exist in this pH range. Hence a very approximate evaluation of *K*_*x*_ was made using lines of slope −3 passing through each separate point of Ce concentration, giving the approximate range of *K*_*x*_ values reported in Table 4 ▸. The values thus obtained are about 18 orders of magnitude lower than the values that can be estimated from Kragten & Decnop-Weever’s (1978 ▸) study in a pH range where Ce(OH)_3_(s) exists.

The activities of Ce(OH)_3_(s) in the solids were calculated by subtracting 20.1 (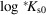) from the *K*_*x*_ values [see equation (11)]. Estimates of the equilibrium activities are also given in Table 4 ▸. Activity coefficients of the solid (λ) were calculated from activity and composition data of the solids by using
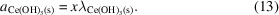
As seen from Table 4 ▸, the values of *K*_*x*_ increase slightly with *x* for the two values we have investigated, showing that they behave thermodynamically as solid solutions. In any case, for both compositions investigated, the concentrations of Ce at equilibrium are lower than those of U in the whole pH range.

It is clear that the determination of the conditional solubility product of Ce(OH)_3_(s) from the coprecipitate and related parameters in a pH range where the pure solid Ce(OH)_3_(s) cannot exist is extremely uncertain and these data are not recommended. They differ also considerably from the data obtained in the higher pH range described below.

**Figure 15:**
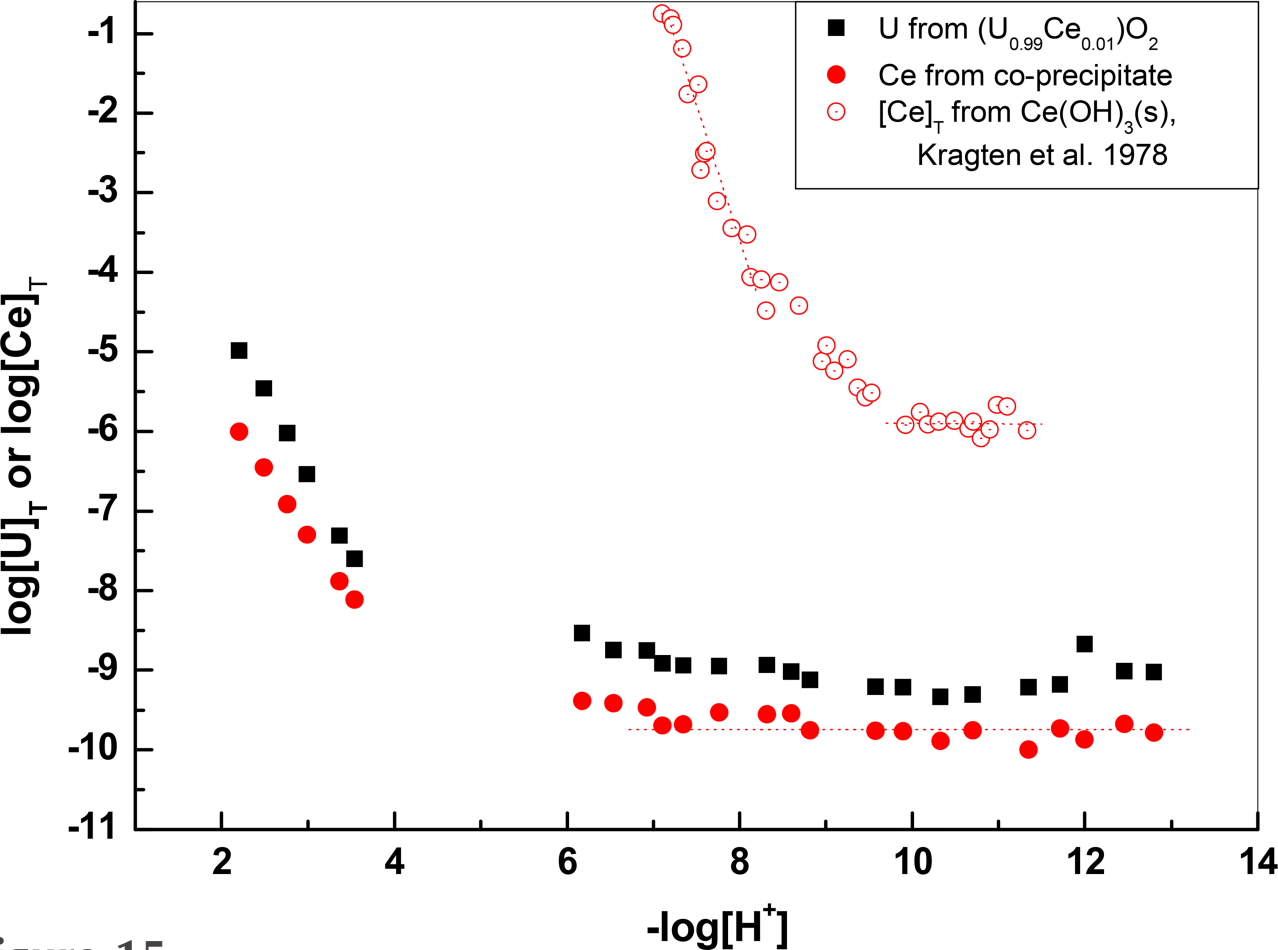
Concentrations of Ce and U in equilibrium with Ce_0.01_U_0.99_ oxide coprecipitate compared with concentrations of Ce in equilibrium with Ce(OH)_3_(s) (Kragten & Decnop-Weever, 1978 ▸) at 1 *M* NaClO_4_.

**Figure 16:**
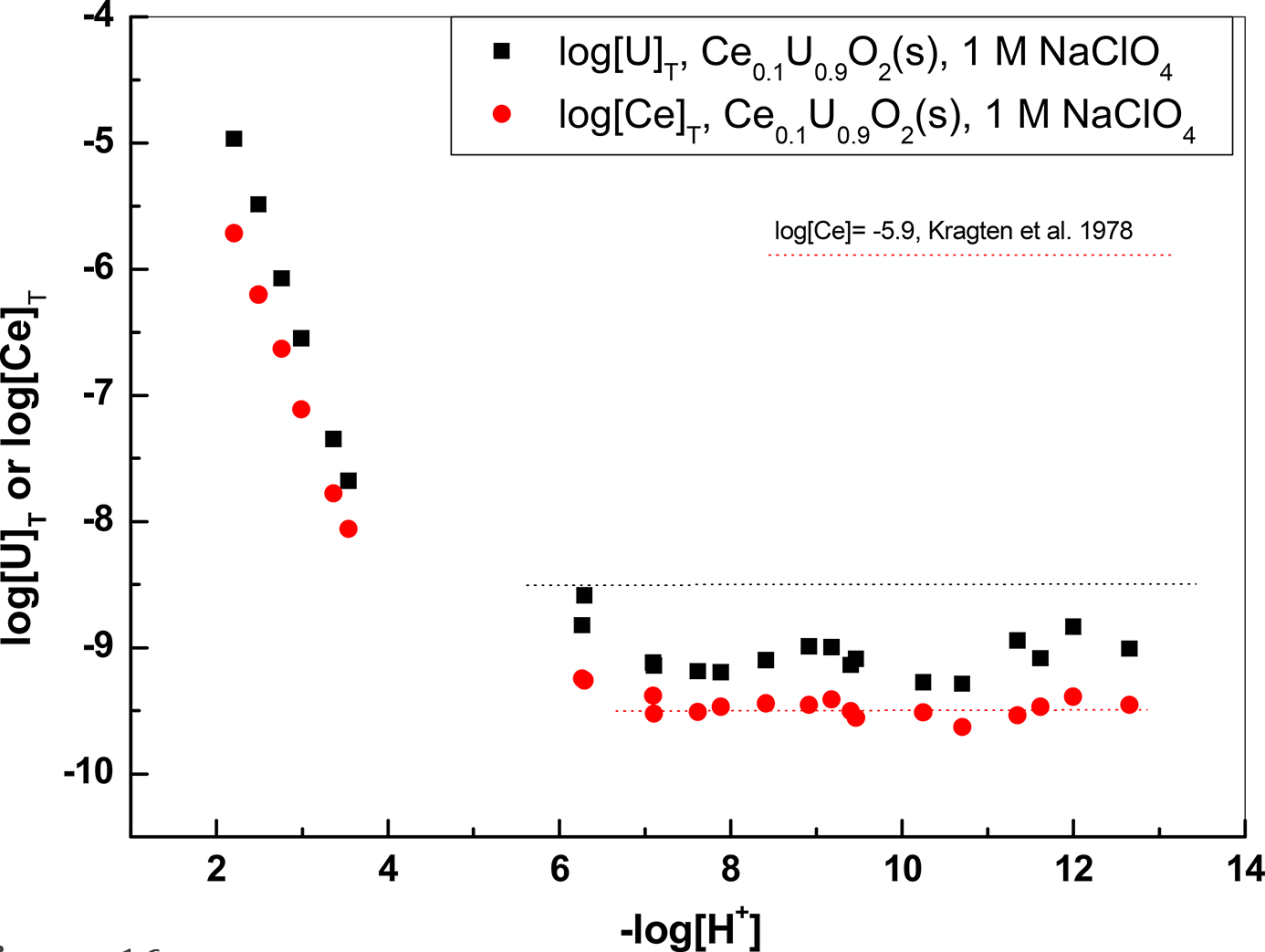
Concentrations of Ce and U in equilibrium with Ce_0.10_U_0.90_ oxide coprecipitate. The dotted lines in the basic range indicate the solubility of UO_2_(s), log[U] = −8.5, and the horizontal part of Ce concentrations for the pure oxide and the coprecipitate.

For −log[H^+^] values higher than 9.9 the solubility of pure Ce(OH)_3_(s) at 1 *M* NaClO_4_ is constant at a value of log [Ce]_T_ = −5.9 due to the dominance of the species Ce(OH)_3_(aq) in solution (see Fig. 15 ▸). In this case, assuming that Ce(OH)_3_(aq) is the major species in solution, we have
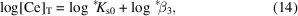
where 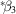 is the constant at 1 *M* NaClO_4_ corresponding to the equilibrium,
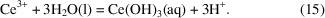
The Ce concentrations in equilibrium with the coprecipitates of Ce with UO_2_ are several orders of magnitude lower than log[Ce] = −5.9 as seen from Figs. 15 ▸ and 16 ▸. This decrease is due to the activity of Ce(OH)_3_(s) in the coprecipitate. In this case we have
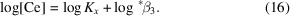


By substituting the value of log 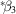 = −26 as determined by Kragten & Decnop-Weever (1978 ▸) in 1 *M* NaClO_4_, we can determine the value of log*K*_0.01_ and log*K*_0.1_ from the Ce concentrations at the high pH range in equilibrium with the corresponding solids. As seen from Figs. 15 ▸ and 16 ▸, the concentrations of Ce at pH > 9.5 are completely determined by congruent dissolution with UO_2_(s) displaying a constant decrease with respect to U concentrations. In Table 5 ▸ are collected the values of *K*_*x*_ determined from Ce concentrations in equilibrium with the coprecipitates for −log[H^+^] > 9.5, together with the activity and activity coefficients λ of Ce(OH)_3_ in the coprecipitates. As seen from Figs. 15 ▸ and 16 ▸, the horizontal part of the Ce (III) concentrations in equilibrium with the coprecipitate stretches even to the −log[H^+^] = 6–9.5 interval, where the solubility of pure Ce(OH)_3_(s) increases steeply due to the presence of Ce(OH)_2_^+^, Ce(OH)^2+^ or Ce^3+^ species. This is a further indication that the release of Ce (III) from the coprecipitate is completely dominated by the release of uranium.

As seen from Table 5 ▸, the values of *K*_*x*_ increase but very slightly with *x* for the two values of *x* investigated here. The negative and much smaller than 1 values of the activity coefficients for Ce(OH)_3_ in the coprecipitate indicate highly favorable mixing properties.

The only reason why the relatively old data of Kragten & Decnop-Weever (1978 ▸) were used in the above discussion is because they seem to be the only data at 1 *M* NaClO_4_ for Ce (III) solubility product and hydrolysis. Another reason was that they used freshly precipitated Ce(OH)_3_(s) like our coprecipitates equilibrated for periods of one week to one month after precipitation. In order to compare the Kragten & Decnop-Weever (1978 ▸) data with more recent reviews of lanthanide hydrolysis, they must be extrapolated to zero ionic strength.

This has been carried out by using the SIT approach (Grenthe *et al.*, 1992 ▸). As a first step, the constants are converted to molal scale, using published density data for NaClO_4_ (Söhnel & Novotny, 1985 ▸) and resulting in *I* = 1.0515 m, Debye–Hückel term *D* = 0.509√*I*/(1 + 1.5√*I*) = 0.2056 and constants in molal scale 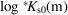 = 20.06 and log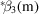 = −25.93. The following SIT equations are valid for the two constants,
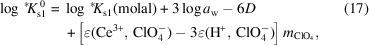

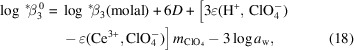
where *a*_w_ = 0.966 is the water activity of 1 *M* NaClO_4_ solutions, the interaction coefficients ɛ (Ce^3+^, ClO_4_^−^) = 0.49 kg mol^−1^ and ɛ(H^+^, ClO_4_^−^) = 0.14 kg mol^−1^.

The resulting value of the solubility product at infinite dilution 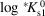 = 18.8 compares relatively well with the value 18.5 ± 0.5 selected in the review of Brown & Ekberg (2016 ▸) and the corresponding value selected for Eu(OH)_3_(am), 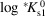 = 16.9 in the review of Jordan *et al.* (2024 ▸), by considering the variation of solubility products of Ln (III) hydroxides with the lattice parameter of hydroxides (Baes & Mesmer, 1976 ▸) as valid.

The value of the constant for the Ce(OH)_3_(aq) 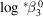 = −24.7 is quite like that reported for Eu(OH)_3_(aq) species (–24.2) by Jordan *et al.* (2024 ▸). Brown & Ekberg (2016 ▸) have not selected values for Ce(OH)_3_(aq) or Eu(OH)_3_(aq) but mention a value 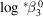 = −24.3 reported by Bernkopf (1984 ▸) for Eu(OH)_3_(aq). An extensive review of Ce-hydrolysis is outside the scope of this work; however, the comparisons above show that the data used are quite reasonable.

The plot of the normalized concentrations of Ce with respect to U concentration has quite some spreads, especially at the low −log[H^+^] range but show that the release of Ce from the solid matrix is totally controlled by uranium dis­solution and does not vary with time, *i.e.* it is a congruent release. The distribution factor according to the Berthelot-Nernst homogeneous distribution law (McIntire, 1963 ▸),
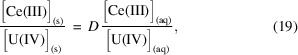
was calculated for both compositions and results in *D* = 0.05 for the Ce_0.01_U_0.99_ solid and *D* = 0.27 for the Ce_0.1_U_0.9_ solid.

## 4. Discussion

The kinetics of the equilibration of the precipitates was relatively fast and in all solubility measurements equilibrium was reached.

The solid characterization results indicate that very probably no phase changes occurred during the equilibration. The chemical composition of the solids was the same within experimental error for the solids before and after equilibration. SEM-EDX mapping of U and Ce shows homogeneous distribution of the components in the solid. The XRD spectra show amorphous solids with quite similar patterns before and after equilibration. The XRD analysis of the solid sample heated to 900°C under reducing atmosphere shows a UO_2_ lattice parameter shrinkage proportional to the Ce (III) content in the solid solution. The lattice parameter change agrees well with those predicted by published relationships for Ce (III) content in stoichiometric U–Ce solids. Vegard’s law, stating that the cell parameter of a solid solution varies linearly with composition between the two end members, cannot be applied directly to such solids because of the hexagonal structure of the Ce_2_O_3_(s) end member. The XPS analysis confirmed Ce (III) in the Ce_0.1_U_0.9_ solid both before and after equilibration, as well as a U:Ce ratio of 9:1, while in the Ce_0.01_U_0.99_ solid Ce it was under the detection limit. The U (IV) oxidation state was confirmed by XPS for all solids, both before and after equilibration.

The results of the EXAFS analysis for U indicating the presence of U (VI) as UO_3_(s) were quite surprising to us. They contradict the solubility results, which show a completely reduced UO_2_(s) phase and the XPS results which show a completely reduced surface. The analysis of the same solid stored in the glove box after dissolving it in HClO_4_ showed the presence of mainly U(IV). The only explanation possible is that the XANES samples were oxidized during transport or at the beamline. It is true that our samples are extremely fine grained, and we did not use an inert atmosphere transport vessel as for the XPS samples. The only potential oxidant during transport, atmospheric oxygen, oxidizes both U (IV) to U (VI) and Ce (III) to Ce (IV) while the XAS analysis indicates reduced Ce (III) and oxidized U (VI). Further, the oxidation of UO_2_(s) by atmospheric oxygen at room temperature is a relatively slow process and UO_2+*x*_ phases are usually formed. There are no other reports of the oxidation of pure UO_2_(s) samples by the XAS beam to our knowledge, and we are not aware of other XAS-EXAFS studies of cerium-doped UO_2_.

The U concentrations in equilibrium with both coprecipitates are in excellent agreement with published data for the solubility of UO_2_(am) indicating that we were successful in maintaining reducing conditions during the tests. In the previous studies of UO_2_(am) coprecipitation with La, Ba and Th by Bruno & Sandino (1987 ▸, 1988 ▸) the concentrations of U at pH 4.5 were higher than 10^−4^ *M* while in the study of Rousseau *et al.* (2002 ▸) they were slightly less than 10 ^−7^ *M* for all pH > 4. No holding reductant was used in these previous studies and electrochemical reduction used, for example, by Russeau *et al.* (2002 ▸) creates very reducing conditions at the electrode surface, but not in the bulk solution.

The measured Ce concentrations in equilibrium with the coprecipitates indicate clearly that the coprecipitates do not behave as homogeneous ideal solid solutions such as, for example, in the case of (U,Np)O_2_ solid solutions studied by Rai *et al.* (2004 ▸). The concentrations of the minor component Ce (III) were totally controlled by the release of U and they were lower than the concentration of U for both Ce_0.01_U_0.99_ solid and Ce_0.1_U_0.9_ solid. In a similar study by Sass & Rai (1987 ▸) of the Cr (III)–Fe (III) hydroxide precipitates, the concentration of Cr (III) is lower than that of Fe (III) for the 1% Cr solid, but already for the 9% Cr solid the concentrations of Cr (III) at equilibrium are higher than those of Fe (III).

## 5. Conclusions

Solids containing two different proportions of Ce (III) and U (IV) were precipitated by carbonate-free NaOH in an Ar glove box atmosphere in the presence of di­thio­nite from acidic solutions. They were equilibrated in 1 *M* NaClO_4_ solutions containing di­thio­nite between pH 2.3 and 12.8 in a glove box atmosphere in undersaturation tests. Experiments were performed for periods up to one month and indicate that equilibrium was achieved relatively fast (less than one week). Several methods were used to characterize the solids (chemical analysis, SEM-EDX, XPS, XAS and EXAFS) confirming homogeneous distribution of Ce in the UO_2_ matrix. The XAS–EXAFS results were the only ones which showed that the samples were completely oxidized to UO_3_(s), apparently by atmospheric oxygen during transport to MAX IV laboratory or by the beam, while Ce was in reduced form as Ce (III). The incorporation of Ce in the UO_2_ solid caused a lattice parameter shrinkage proportional to the Ce content.

The solubility of the coprecipitates was determined in the pH range 2.2–12.8 in 1 *M* NaClO_4_ solutions in an Ar glovebox and in the presence of di­thio­nite. The U concentrations were in excellent agreement with the lower limit of the UO_2_(am) solubilities selected by NEA-TDB (Grenthe *et al.*, 2020 ▸), as expected for coprecipitates with relatively low Ce content. The Ce concentrations were completely dominated by the release of U and were lower by about an order of magnitude than those of U over the whole pH range studied. The Ce concentrations increase slightly with the increase of Ce content in the solid, suggesting that Ce_*x*_U_1–*x*_O_2±*y*_ solids behave thermodynamically as solid solutions. The conditional solubility product of Ce(OH)_3_ from the coprecipitate was several orders of magnitude (∼4 in the near neutral pH range and ∼18 in the acidic range) lower than that of pure Ce(OH)_3_(s). The activity coefficients of Ce(OH)_3_(s) in the coprecipitate are much less than 1 indicating that the mixing of Ce(OH)_3_ with UO_2_ is highly favorable.

These results indicate that the concentrations of Ce, other lanthanides and fission products released by the fuel matrix during oxidative dissolution will not be determined by their individual solubilities when they coprecipitate with UO_2_(s) at the iron surface of the canister insert but will be orders of magnitude lower. The major goal of this study was to refine the techniques and procedures for maintaining appropriate reducing conditions. The initial results, when we relied too much only on the high concentration of di­thio­nite present in our solutions and carried measurements in presence of air, gave two to three orders of magnitude higher U concentrations. Only after taking the special precautions described in the experimental part of the manuscript (degassing all solutions, working in a glove box with *p*_O2_ < 1 p.p.m., filling the tubes almost to the top *etc*.) were we able to obtain reliable data. This experience will be very valuable when we undertake the study of coprecipitation of UO_2_ with other actinides.

## Figures and Tables

**Figure 4:**
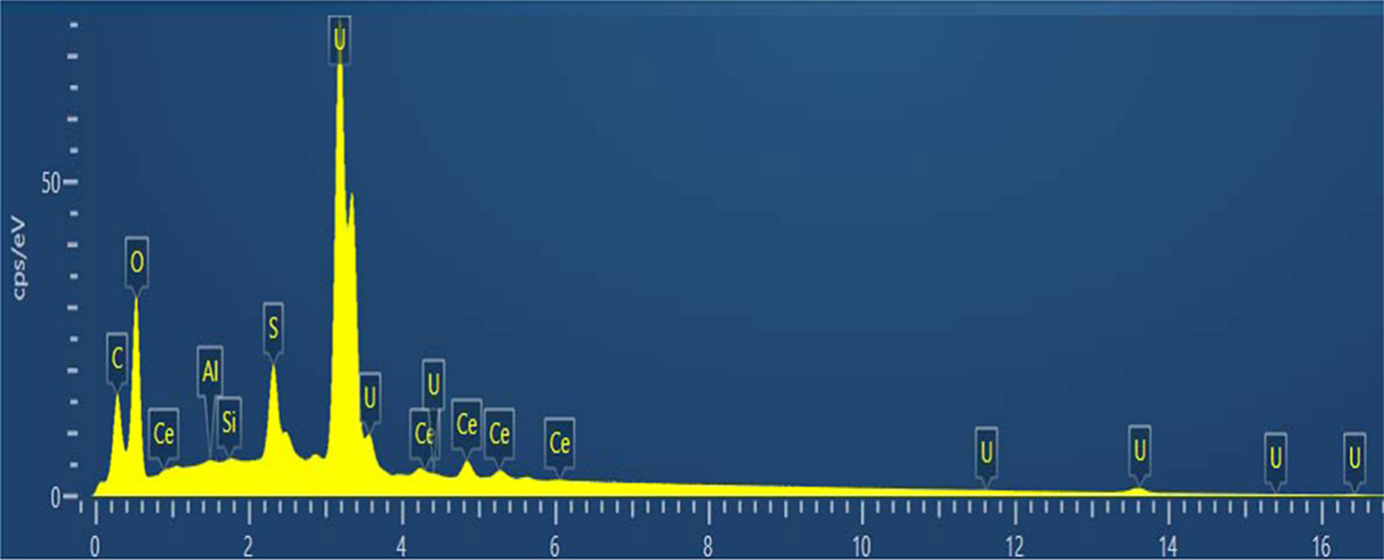
SEM-EDX spectrum of the Ce_0.1_U_0.9_ solid. Solid after equilibration; S originates from di­thio­nite.

**Table 1:** Analytical molar fractions of U and Ce in the initial and equilibrated solids

Target composition	Before equilibration		93 days of equilibration	
Ce_*x*_U_1–*x*_	Ce	U	Ce	U
Ce_0.01_U_0.99_	0.01	0.99	0.01	0.99
Ce_0.10_ U_0.90_	0.09	0.90	0.09	0.90

**Table 2:** Lattice parameters for the Ce (III)–U (IV) coprecipitates sintered at 900°C

Composition	Expected from equation (3)	*a* (Å) refined
UO_2.00_	–	5.47127
Ce_0.01_U_0.99_	5.4707	5.4709
Ce_0.09_ U_0.90_	5.4655	5.4660

**Table 3:** Mean bond distances *d* (Å), Debye–Waller factors σ^2^, number of distances *N*, shift in the threshold energy Δ*E*_0_ (eV), amplitude reduction coefficient 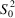, and error square sum as defined in the *EXFSPAK* program package *F* of solid ‘UO_2_’ samples as determined by EXAFS at room temperature

Interaction	*N*	*d*	σ^2^	Δ*E*_0_	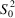	*F*
Sample ‘UO_2__pure_1’, *k* = 2.0–13.0 Å^−1^						
U=O	2	1.782 (1)	0.0051 (1)	−9.2 (2)	0.696 (8)	16.74
MS (O=U=O)	3*2	3.564	0.0057 (12)			
U—O	4	2.319 (3)	0.0155 (3)			
U⋯U	4	3.414 (6)	0.0206 (7)			
U⋯U	4	4.122 (6)	0.0245 (8)			

Sample ‘UO_2__pure_2’, *k* = 2.0–13.0 Å^−1^						
U=O	2	1.789 (1)	0.0049 (1)	−6.4 (2)	0.694 (10)	20.25
MS (O=U=O)	3*2	3.578	0.0026 (5)			
U—O	4	2.337 (3)	0.0157 (4)			
U⋯U	4	3.51 (2)	0.022 (2)			
U⋯U	4	4.191 (7)	0.0231 (9)			

Sample ‘U_0.99_Ce_0.01_’, *k* = 2.0–13.0 Å^−1^						
U=O	2	1.784 (1)	0.0044 (1)	−6.5 (2)	0.656 (7)	15.80
MS (O=U=O)	3*2	3.566	0.0015 (6)			
U—O	4	2.349 (2)	0.0154 (3)			
U⋯U	4	3.483 (7)	0.0232 (9)			
U⋯U	4	4.168 (5)	0.0212 (5)			

Sample ‘U_0.90_Ce _0.1_’, *k* = 2.0–13.0 Å^−1^						
U=O	2	1.788 (1)	0.0056 (1)	−6.9 (2)	0.700 (8)	14.18
MS (O=U=O)	3*2	3.576	0.0058 (9)			
U—O	4	2.328 (3)	0.0206 (4)			
U⋯U	4	3.500 (12)	0.032 (2)			
U⋯U	4	4.168 (3)	0.0191 (3)			

**Table 4:** Values of log *K*_*x*_, 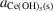 and 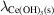 estimated from data in the −log[H^+^] range 2.2–3.6

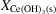	log *K*_*x*_	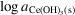	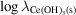
1	20.1	0.00	0.00
0.1	2.5 ± 0.7	−17.6	−16.6
0.01	2.3 ± 0.7	−17.8	−15.8

**Table 5:** Values of log *K*_*x*_, *a*_Ce(OH)3(s)_ and λ_Ce(OH)3(s)_ estimated from data −log [H^+^] > 9.5

X_Ce(OH)3_	log *K*_*x*_	log *a*_Ce(OH)3(s)_	log λ_Ce(OH)3(s)_
1	20.1	0.00	0.00
0.1	16.5	−3.6	−2.6
0.01	16.3	−3.8	−1.8

## Data Availability

Data will be made available on request.
